# Cellular uptake and in vivo distribution of mesenchymal-stem-cell-derived extracellular vesicles are protein corona dependent

**DOI:** 10.1038/s41565-023-01585-y

**Published:** 2024-02-16

**Authors:** Revadee Liam-Or, Farid N. Faruqu, Adam Walters, Shunping Han, Lizhou Xu, Julie Tzu-Wen Wang, Jennifer Oberlaender, Alberto Sanchez-Fueyo, Giovanna Lombardi, Francesco Dazzi, Volker Mailaender, Khuloud T. Al-Jamal

**Affiliations:** 1 Institute of Pharmaceutical Science, Faculty of Life Sciences & Medicine, King’s College London, London, UK; 2 Pharmacology Department, Faculty of Medicine, University of Malaya, Kuala Lumpur, Malaysia; 3 Max Planck Institute for Polymer Research, Mainz, Germany; 4 Department of Dermatology, University Medical Center of the Johannes Gutenberg-University Mainz, Mainz, Germany; 5 Institute of Liver Studies, King’s College London University and King’s College Hospital, London, UK; 6 Peter Gorer Department of Immunobiology, School of Immunology and Microbial Sciences, King’s College London, London, UK; 7 Comprehensive Cancer Centre, King’s College London, London, UK

**Keywords:** Nanoparticles, Drug delivery

## Abstract

Extracellular vesicles (EVs) derived from mesenchymal stem cells are promising nanotherapeutics in liver diseases due to their regenerative and immunomodulatory properties. Nevertheless, a concern has been raised regarding the rapid clearance of exogenous EVs by phagocytic cells. Here we explore the impact of protein corona on EVs derived from two culturing conditions in which specific proteins acquired from media were simultaneously adsorbed on the EV surface. Additionally, by incubating EVs with serum, simulating protein corona formation upon systemic delivery, further resolved protein corona–EV complex patterns were investigated. Our findings reveal the potential influences of corona composition on EVs under in vitro conditions and their in vivo kinetics. Our data suggest that bound albumin creates an EV signature that can retarget EVs from hepatic macrophages. This results in markedly improved cellular uptake by hepatocytes, liver sinusoidal endothelial cells and hepatic stellate cells. This phenomenon can be applied as a camouflage strategy by precoating EVs with albumin to fabricate the albumin-enriched protein corona–EV complex, enhancing non-phagocytic uptake in the liver. This work addresses a critical challenge facing intravenously administered EVs for liver therapy by tailoring the protein corona–EV complex for liver cell targeting and immune evasion.

## Abstract (web summary)

In regenerative medicine, stem-cell-derived extracellular vesicles are emerging as cell-free nanotherapeutics. Here, the authors show that coating these nanovesicles with blood proteins such as albumin improves their uptake by liver cells, offering a better treatment strategy for liver diseases.

## Main

Although chronic liver diseases can be caused by a variety of different aetiologies, they are all characterized by a continuous process of inflammation, oxidative stress and scarring, which concur in promoting liver deterioration, cirrhosis and ultimately liver failure and death^1^. This pathophysiological progression is underpinned by a variety of different liver cell subsets, including hepatocytes, hepatic stellate cells, liver sinusoidal endothelial cells (LSECs), macrophages and lymphoid cells. Developing a broad-spectrum treatment that can be utilized to ameliorate a range of liver diseases requires therefore the potential to target different liver cell populations^2,3^.

There is a growing body of evidence suggesting that the immunomodulatory and rejuvenative properties of mesenchymal stem cells (MSCs) are mediated through the paracrine effects of the extracellular vesicles (EVs) they secrete^4^. In preclinical models of liver injury, studies have demonstrated that MSC-derived EVs (MSC EVs) can reduce collagen deposition and hepatic inflammation^5^, suppress hepatic stellate cell activation^6^, promote hepatocyte proliferation^7^ and inhibit hepatocyte apoptosis^8^. The use of EVs, as opposed to MSCs, as therapeutic agents brings many additional benefits including feasibility of large-scale production, the ease of storage and batch-to-batch quality control. Unlike cell-based approaches, EVs may also be engineered, for example, by surface modification, to improve pharmacokinetic and pharmacodynamic properties^9^.

We have previously shown that EVs from cancer and non-cancer cell origin can accumulate in the liver after intravenous administration although their cellular uptake profile remains unknown^10,11^. Recently, it has been shown that the so-called ‘protein corona’ can greatly affect the behaviour of synthesized particulates in vivo, including cellular targeting^12,13^. Nevertheless, the effects of protein corona on the in vivo fate of EVs have never been unravelled^14^.

The protein corona is formed when nanoparticles (NPs) come into contact with biological fluids, and proteins are adsorbed onto the NP surface. In contrast to synthetic NPs, culture-derived EVs encounter proteins at two time points: first, during the production in the cell culture condition; and second, on injection. Their corona can therfore be composed of both a ‘primary’ and ‘secondary’ corona formed at the production site and on contact with bodily fluids, respectively. The relative contribution of these two coronas in terms of particle properties and in vivo behaviour has yet to be described.

In this study, we aimed to determine how different good manufacturing practice (GMP)-compatible culturing methods affect EVs’ corona compositions and subsequently impact of liver cell targeting and accumulation, and to identify the key proteins mediating the in vivo behaviour of EVs. We hypothesize that the corona is composed, in part, of proteins derived from the cell culture media and the in vivo environment upon administration. MSC EVs were isolated from two-dimensional (2D) (EV_2D_) and 3D (EV_3D_) cultures^15^. Comparative proteomic analysis and measurement of in vivo organ biodistribution and uptake of EVs in liver cell subpopulations were undertaken. Principal component analysis (PCA) was applied to correlate corona compositions with biological functions.

## Results

### EVs from 2D and 3D cultures are physically comparable

**Fig. 1:**
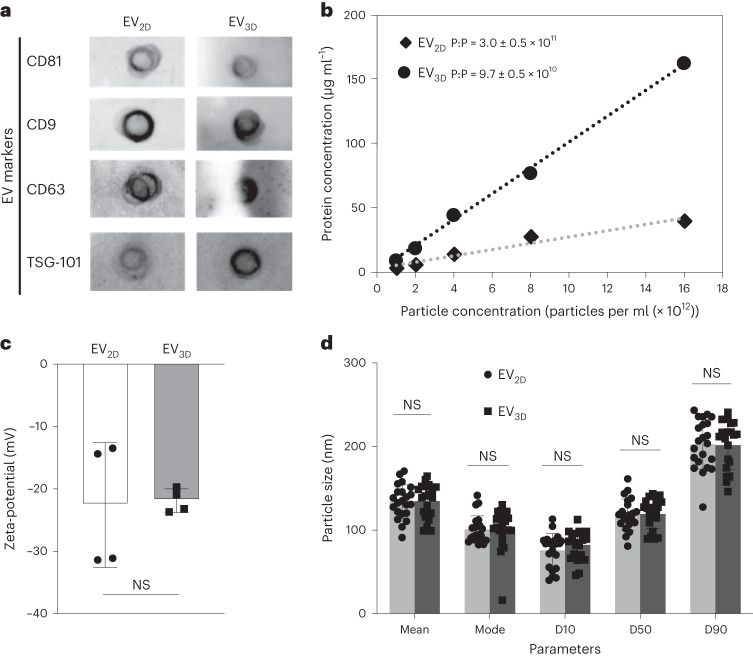
Physicochemical and biochemical characterization of EV_2D_ and EV_3D_. **a**, Expression of EV surface markers (CD81, CD9, CD63) and internal marker (TSG101) analysed by chemiluminescence dot-blot. Equal numbers of EVs (5 × 10^10^ particles per ml) were spotted on the nitrocellulose membrane prior to staining. **b**, Protein concentration measured by microBCA assay correlated with particle concentration measured by NTA (*n* = 5). **c**, Zeta-potential of EVs in deionized water (*n* = 4, biologically independent samples). Data are presented as mean ± s.d. **d**, Size of EVs in PBS measured by NTA (*n* = 22, biologically independent samples). Percentiles (D10, D50 and D90) determine particle size distribution. EV_2D_ and EV_3D_ exhibited comparable physicochemical and biochemical characteristics except for protein content (EV_3D_ > EV_2D_). Data are presented as mean and mode ± s.d. Statistical analysis was performed by two-tailed unpaired *t*-test: NS, not significant, *P* > 0.05. Source data

Serum-free conditioned culture media (CCM) and KnockOut serum replacement (KO)-containing CCM derived from 2D and 3D MSC cultures, respectively, were subjected to EV isolation. Dot-blot analysis (Fig. 1a) shows that EV_2D_ and EV_3D_ were positive for canonical tetraspanins, that is, CD81, CD9 and CD63. The expression of an endosome-associated protein, TSG101, in both EVs confirms their endosomal origin^15^. The null microBCA read-outs of isolated unconditioned media for 2D and 3D culture confirmed that free proteins present in the media could not be co-isolated (Supplementary Fig. 1). The measurement of total EV protein content correlated with the particle concentration, as determined by micro bicinchoninic acid assay (microBCA) and nanoparticle tracking analysis (NTA), respectively (Fig. 1b). EV_3D_ had a higher protein content than EV_2D_ in agreement with the threefold increase in particle to protein ratio (P:P) achieved for EV_2D_ (3.0 ± 0.5 × 10^11^) versus EV_3D_ (9.7 ± 0.5 × 10^10^), respectively, both of which were within the acceptable purity range reported^16^. EV_2D_ and EV_3D_ were identical in surface charge as determined by zeta-potential values (Fig. 1c) and size (Fig. 1d), determined by mean, mode and particle size distribution (see Supplementary Fig. 2 for representative NTA and transmission electron microscopy results), indicating the acceptable EV charge/size range obtained^17,18^.

### Culturing conditions affects protein corona identity of EVs

To mimic the EV–serum protein interaction in vivo, EV_2D_ and EV_3D_ were exposed to 50% (v/v) EV-depleted fetal bovine serum (FBS) (EV-D FBS) and subjected to ultracentrifugation and protein corona desorption to obtain the EV with bound hard corona (HC-EV_2D_ and HC-EV_3D_), hard corona (that is, protein associated with EV following stripping, HC_2D_ and HC_3D_), and stripped EVs (strip-EV_2D_ and strip-EV_3D_) (see Supplementary Fig. 3 for procedure). The protocol was optimized to efficiently desorb HC (Supplementary Tables 1 and 2 and Supplementary Fig. 4).

**Fig. 2:**
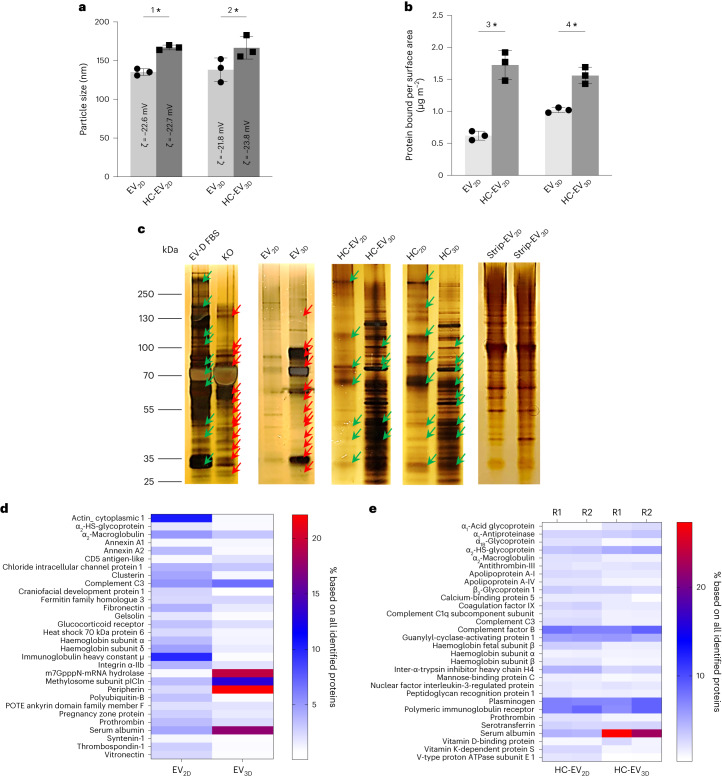
Protein corona formation on EVs derived from different culturing conditions evaluated by NTA, microBCA assay, SDS–PAGE and LC–MS. **a**, Size changes due to protein corona formation (*n* = 3; ^1^*P = 0.014, ^2^*P = 0.019). **b**, Protein bound per surface area of EVs measured by microBCA assay (*n* = 3; ^3^*P = 0.012, and *^4^*P* = 0.031). Data are presented as mean ± s.d. Statistical analysis is performed using two-tailed paired *t*-test (*P < 0.05). **c**, Representative silver-stained SDS–PAGE of EVs (*n* ≥ 2), HC-EV, HC and stripped EVs (strip-EV). EV-D FBS and KO are FBS incubation controls and culture media, respectively. Similarities and differences are depicted as green and red arrows, respectively. The results confirmed HC formation of both EV types although the compositions are qualitatively different. **d**,**e**, Quantitative LC–MS analysis of protein corona formation. The most abundant proteins in the non-FBS-incubated (**d**) and FBS-incubated (**e**) EVs identified by LC–MS against human protein and bovine protein databases, respectively, are displayed in the heatmap (R1 and R2 represent two biological replicates). Both EV_3D_ and HC-EV_3D_, unlike their 2D counterpart, are rich in albumin. Values are expressed as percentage abundance of total protein amounts identified (*n* = 2, biological replicates with *n* = 3, technical replicates per sample). Source data

As shown in Fig. 2a, after the removal of unbound FBS proteins, the HC-EV_2D_ and HC-EV_3D_ were found to be significantly larger in size than non-incubated EV. Despite this, no significant change in zeta-potential could be observed. The protein bound per surface area measured by microBCA assay was also found to be increased, indicating the additional protein was acquired from EV-D FBS (Fig. 2b). Visualization of protein bands was carried out by loading digested EV, HC-EV, HC, stripped EV, EV-D FBS and KO on sodium dodecyl sulfate–polyacrylamide gel electrophoresis (SDS–PAGE) gels and performing silver staining (Fig. 2c). KO and EV-D FBS were included to determine which proteins were acquired from FBS incubation compared to those intrinsically found in EVs. Distinct protein profiles between EV_2D_ and EV_3D_ can be observed, with EV_3D_ showing similarities to KO medium proteins which were absent in EV_2D_. This finding was confirmed to be independent of the cells’ geometry (Supplementary Fig. 5). Upon exposure to EV-D FBS, an HC is formed on the EV surface, causing the protein adsorption on the EV with different preferential compositions when comparing HC-EV_2D_ and HC-EV_3D_. Similar protein patterns were also observed in HC_2D_ and HC_3D_ bands. The differences in protein bands disappeared in the stripped EV_2D_ and stripped EV_3D_ samples. Altogether, the results suggest that culturing supplementation affects the protein identity of EVs and the protein corona formed upon exposure to exogenous proteins, that is, from serum. To distinguish the type of corona formed, we refer to the protein corona acquired from CCM and FBS incubation as primary and secondary corona, respectively.

Proteomic analysis of EV_2D_ and EV_3D_ was performed by liquid chromatography–mass spectrometry (LC–MS) and analysed using the human protein database. The top 31 identified proteins expressed as a relative protein abundance in percentage (%RPA) are displayed in the heatmap as shown in Fig. 2d (see Supplementary Table 3 for full details). The differences in the protein enrichment of EV_2D_ and EV_3D_ indicate that although EVs are secreted from the same parental cells, differential culturing conditions significantly affect their protein profiles. As EV_2D_ were isolated from serum-free media, it is expected that the proteins detected in EV_2D_ will be free from cell-culture supplements. The most abundant proteins found in EV_2D_ were actin cytoplasmic 1 and immunoglobulin heavy constant mu, which are reported as subcellular components and secretomes of MSCs, respectively^19,20^. In contrast, the EV_3D_ media had to be supplemented with KO to maintain the spheroidal condition. Three protein components, that is, serum albumin, m7GpppN-mRNA hydrolase (DCP2), methylosome subunit pICln (CLNS1A) and peripherin (PRPH) were the most abundant in EV_3D_. DCP2, CLNS1A, and PRPH are identified as MSC cytoplasmic proteins, whereas only albumin does not originate from MSCs and is classified as secreted protein^21–23^. It was therefore suggested that albumin was derived from the KO supplement and formed as the first corona during culture. This is also confirmed by comparing the LC–MS data of EV_3D_ and KO supplement (Supplementary Fig. 6). The protein profile of HC adsorbed on the EVs after exposure to FBS was analysed against a bovine protein database to exclude all human-derived proteins in the samples and focus on the protein layer obtained from FBS incubation only (that is, the second corona). Figure 2e shows the heatmap of the top 30 identified proteins as %RPA (Supplementary Table 4 for full details). The differences in HC profile reveal that differential culturing conditions also affect the protein compositions of the second corona. Bovine-derived albumin (Supplementary Fig. 7) was found to be prominently enriched on EV_3D_ compared with EV_2D_. Altogether, the LC–MS data support the previous silver-stained SDS–PAGE results and suggest that the culturing conditions of 3D culture enabled the formation of the first and second coronas enriched with albumin on EV_3D_.

### Protein corona modulates cellular uptake in vitro

**Fig. 3:**
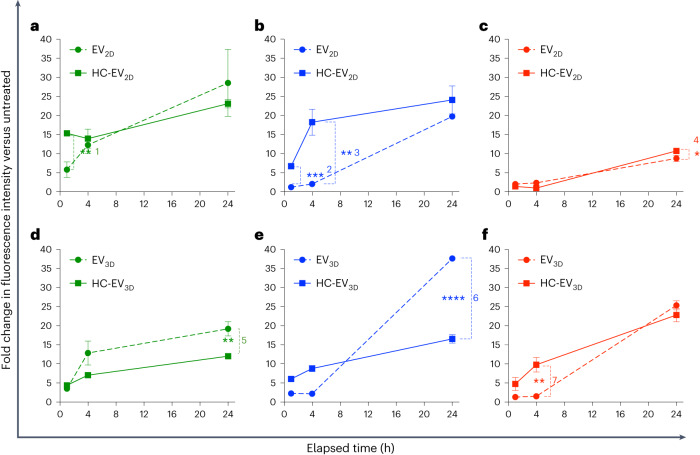
Cellular uptake of non-incubated EVs and HC-EVs in phagocytic and HepG2 cells. **a–f**, Cellular uptake of EV_2D_ ± HC (**a**–**c**) and EV_3D_ ± HC (**d**–**f**) in phagocytic J774 cells (**a**,**d**), human-monocyte-derived macrophages (Hu-Ø) (**b**,**e**) and HepG2, representing non-phagocytic liver hepatocytes (**c**,**f**). AF488-labelled EVs or HC-EVs were incubated with cells at a dose of 2 × 10^9^ particles per well (24-well plate) for 1 h, 4 h and 24 h. Cellular uptake was measured by flow cytometry, and uptake was expressed as fold increase of the mean AF488 signal per cell (MFI) compared with non-treated cells. Doping of EVs with albumin-rich HC, that is, the case of EV_3D_, significantly reduces uptake in phagocytic cells while keeping uptake in HepG2 cells unchanged (^1^**P = 0.00417, ^2^***P = 0.00021, ^3^**P = 0.00243, ^4^*P = 0.04176, ^5^**P = 0.00871, ^6^****P = 0.00003, ^7^**P = 0.00522). Data are presented as mean ± s.d. (*n* = 3) with two-tailed unpaired *t*-test (*P < 0.05, **P < 0.01, ***P < 0.001, ****P < 0.0001). Source data

Because the EVs in this study were prepared with the aim of increasing the accumulation in liver cells other than phagocytic cells, the uptake of EVs (HC-free EVs and HC-coated EVs) by macrophages and liver parenchymal cells were in vitro simulated in phagocytic cells, that is, J774 and human-monocyte-derived macrophages (Hu-Ø) and human hepatocellular carcinoma cell line, HepG2, respectively, for 1 h, 4 h and 24 h. Uptake was evaluated by the fold increase in the mean fluorescence intensity signal per cell (MFI) compared with non-treated cells (see Supplementary Figs. 8 and 9 for labelling rationale). The presence of HC on EV_2D_ significantly increased the uptake by phagocytic cells during 1 h incubation in J774 and 1 and 4 h incubation in Hu-Ø (Fig. 3a,b), respectively, while the increase in uptake by HepG2 was observed only at 24 h incubation (Fig. 3c). In the case of HC-EV_3D_, a significant reduction of phagocytic uptake at 24 h incubation was observed compared with EV_3D_ (Fig. 3d,e). In HepG2, a significant increase in EV_3D_ uptake mediated by HC was observed at 4 h incubation. Remarkably, both EV_3D_ and HC- EV_3D_ could be preferentially taken up by HepG2 compared with their 2D counterparts. This preferential behaviour could also be observed when labelling EVs by lipophilic dye incorporation (Supplementary Fig. 10). Altogether, the results suggest that HC-EV_3D_ shows favourable uptake profiles by the liver microenvironment indicated by reduced and increased uptake by phagocytic cells and hepatocytes, respectively.

### Non-phagocytic liver cells show preference for EV_3D_ in vivo

**Fig. 4:**
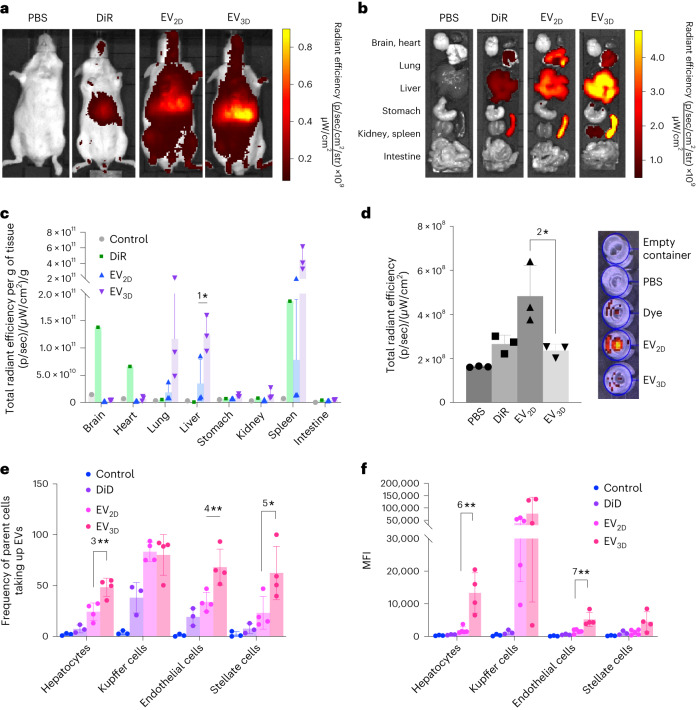
In vivo organ biodistribution and cellular uptake by liver subpopulations of EVs. **a**–**d**, Animals were intravenously injected with 2 × 10^11^ DiR-labelled EVs, PBS or the free dye (control): representative whole-body live (ventral) imaging (**a**), ex vivo images of whole major organs (**b**), semiquantitative analysis of the ex vivo images (*n* = 3 for EV samples, ^1^*P = 0.0464) (**c**) and urine clearance at 24 h post-injection (*n* = 3, ^2^*P = 0.0393) (**d**). Fluorescence intensity measured as total radiant efficiency per gramme of tissue was obtained by using an ROI tool and Living Image v.4.7.3 software. **e**,**f**, Cellular uptake of EVs by liver subpopulations (hepatocytes, Kupffer cells, endothelial cells and stellate cells) by flow cytometry shown as cell number positive for the signals (*n* ≥ 3, ^3^**P = 0.0032, ^4^**P = 0.0089 and ^5^*P = 0.0371) (**e**) or MFI (n ≥ 3, ^6^**P = 0.0014 and ^7^**P = 0.0033) (**d**). EV_3D_ showed significantly higher uptake in hepatocytes, endothelial cells and stellate cells but not Kupffer cells. Data are presented as mean ± s.d. with two-tailed unpaired *t*-test (**c**,**d**) and one-way ANOVA with post hoc Tukey test (**e**,**f**) (*P < 0.05, **P < 0.01). Source data

A biodistribution study was carried out in mice to determine if the differences found in vitro prevail in vivo. DiR-labelled EV_2D_ and EV_3D_ were intravenously administered for optical imaging (see Supplementary Fig. 8d for labelling and Supplementary Table 5 for dose information). At 24 h post-injection, whole-body imaging was performed as shown in Fig. 4a (see Supplementary Fig. 11 for 1 and 4 h biodistribution). The brightest signals could be detected in the upper abdominal area, corresponding to the location of the liver and spleen, with mice injected with EV_3D_ illustrating the highest DiR signal. This is supported by the ex vivo imaging of major organs, which revealed that EV_3D_ accumulated most in the liver and spleen over a 24 h period (Fig. 4b). Semiquantitative analysis of ex vivo organ images (normalized to organ weights) was carried out (Fig. 4c) and suggested that both EVs recorded the highest accumulation in the spleen and liver, followed by lungs and kidneys. When comparing the accumulations between two types of EVs in the liver, higher DiR signals were detected in the EV_3D_ group (*P* < 0.05), agreeing with the in vivo imaging. Mice were also individually housed in a metabolic cage for urine correction to check for renal clearance (Fig. 4d). Interestingly, the highest DiR signals in the urine were detected in the EV_2D_ group, confirming the shorter half-life of EV_2D_ in the circulation system.

To investigate in vivo uptake by liver subpopulations, that is, hepatocytes, Kupffer cells, endothelial cells and stellate cells, DiD-labelled EV_2D_ and EV_3D_ were intravenously administered (see Supplementary Table 5 for dose information). At 24 h post-injection, mice were anaesthetized, followed by liver perfusion (see Supplementary Fig. 12 for liver perfusion procedure). Digested livers were subjected to differential centrifugation to isolate hepatocytes and non-parenchymal cell (NPC) population (that is, Kupffer cells, endothelial cells and stellate cells) (see Supplementary Fig. 13 for isolation procedure)^5^. Cell fractions were then stained with fluorescently conjugated antibodies for the markers reportedly expressed by these subpopulations to enable cell type identification co-localized with DiD signal of EVs by flow cytometry (see Supplementary Fig. 14 for antibody panel and gating strategy). Liver subpopulations could be isolated with consistency in numbers (Supplementary Fig. 15). DiD signal was also co-localized with the signal of anti-human anti-CD9 antibody in hepatocytes, confirming the labelling stability of EVs in vivo (Supplementary Fig. 16). As shown in Fig. 4e,f, the majority of Kupffer cells (∼90%) took up EVs with no preference between EV_2D_ and EV_3D_, while differences could be observed in the other cell subpopulations: 48.38% of hepatocytes, 68.15% of endothelial cells and 62.38% of stellate cells were positive for EV_3D_ with mean fluorescence intensity (MFI) values of 13,338.5, 5,219.75 and 4,478, respectively. Values were significantly reduced for EV_2D_ samples with percentage of positive cells and MFI values of 24.25%, 33.88%, 23.15% and 1,868, 1,538, 1,237, obtained for hepatocytes, endothelial cells and stellate cells, respectively. In summary, macrophages are indifferent to EV type for uptake, whereas hepatocytes and endothelial cells, followed by stellate cells appeared to favour uptake of EV_3D_ over EV_2D_ which could explain the overall higher liver signal for EV_3D_.

### EV opsonization is predictably protein corona dependent

To understand if there is a correlation between EVs’ in vitro and in vivo behaviours, biological processes and protein corona compositions, EV and HC proteins identified by LC–MS were subjected to Gene Ontology (GO) analysis. To focus on EV proteins potentially playing a role in cellular uptake, only identified proteins coded for extracellular space with highly significant enrichment (*P* < 0.001, Supplementary Fig. 17) were selected for further analysis (see Supplementary Table 6 for protein accession codes).

**Fig. 5:**
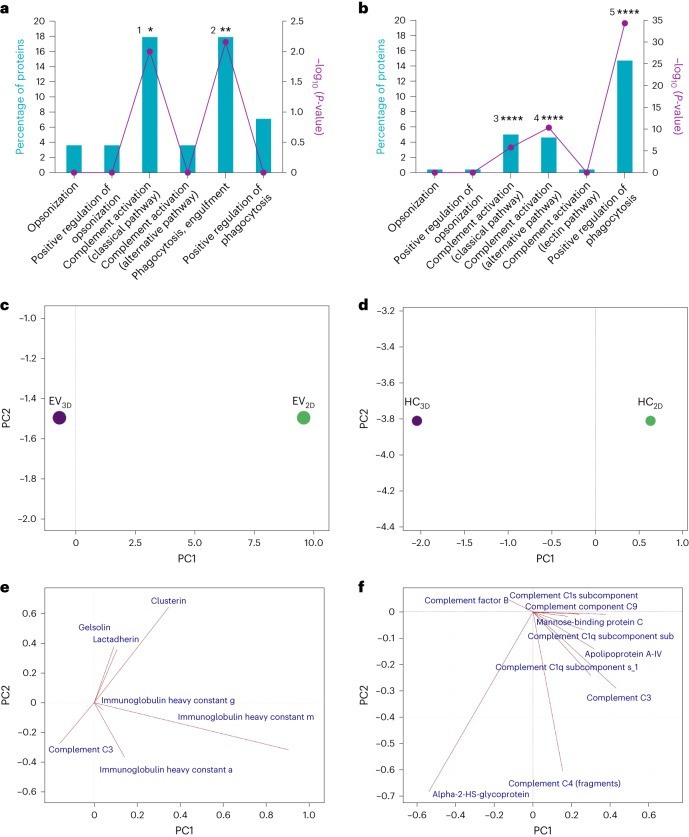
GO analysis for classification of the protein identified by LC–MS. **a**,**b**, Proteins detected in both EV_3D/2D_ and HC_2D/3D_ were decoded to obtain a gene list involved in biological processes contributing to the clearance of non-incubated EVs (against in-built UniProt human database, GO analysis with Bonferroni correction (^1^*P = 0.01 and ^2^**P = 0.007) (**a**) and hard protein corona of incubated EVs (against in-built UniProt non-human mammal (bovine) database, GO analysis with Bonferroni correction, ^3^****P = 1.6 × 10^−6^, ^4^****P = 4.97 × 10^−11^ and ^5^****P = 4.89 × 10^−35^) (**b**). **c**,**d**, GO analysis was performed using FunRich software v.3.1.3. Significantly enriched proteins (hypergeometric and Bonferroni analysis *P < 0.05, **P < 0.01 and ****P < 0.0001) by quantity underwent PCA for separation of EV_2D_ and EV_3D_ (**c**) and HC_2D_ and HC_3D_ (**d**), shown as score plots. **e**,**f**, Loading plots were used to illustrate how each protein influences the computed PCs in **c**,**d**, respectively. Overall, the intrinsic properties of EV protein corona constituents are fundamentally different. HC_2D_ proteins are associated with higher extents of complement activation. The PCA was performed using mean of the LC–MS data (two biological and three technical replicates), and EVs were pooled from three batches. Source data

Subsequent enrichment analysis was then performed by biological processes for both EV and HC sets against the human and bovine databases (UniProt), respectively. Proteins related to significantly enriched processes (Fig. 5a,b) were quantitatively analysed by PCA to obtain a set of new variables called principal component (PC) corresponding to a linear combination of the originals. The score plot as shown in Fig. 5c,d displayed a difference between EVs and HCs by considering only PC1, respectively (see Supplementary Fig. 18 for scree plot). When examining the loading plots to identify variables with the highest impact of each component, the immunoglobulin heavy constant mu strongly influenced the separation between EV_2D_ and EV_3D_ on PC1 (Fig. 5e). The higher abundance of this protein on EV_2D_ (see Supplementary Fig. 19a for Bi-plot) made them favoured for engulfment by phagocytes and to induce complement activation (classical pathway) as interpreted from associated protein functions (UniProt database). The loading plots of HC samples (Fig. 5f) suggest the separation of HC_2D_ and HC_3D_ by α-2-HS-glycopotein is more abundant in HC_3D_. The second most influential protein was complement C3 which shows collinearity with various proteins that play a major role in the EV clearance via several processes. Supplementary Tables 3 and 4 display protein contents and Supplementary Tables 7 and 8 show the contribution to the biological processes. The higher abundance of these highly related proteins in HC_2D_ (see Supplementary Fig. 19b for Bi-plot) suggests that upon systemic exposure EV_2D_ clearance is promoted via opsonization, complement activation and phagocytosis^24–26^.

### Albumin contributes to uptake of EVs in liver cells in vivo

We hypothesized that the presence of albumin in the first corona acts as an attraction site for additional albumin molecules to adsorb onto the EV surface to form the second corona layer. We further hypothesized that these albumin molecules in the HC potentially increase the uptake in liver cells due to the abundance of albumin receptors and their subtype (SPARC) (Supplementary Fig. 20).

**Extended Data Fig. 1:**
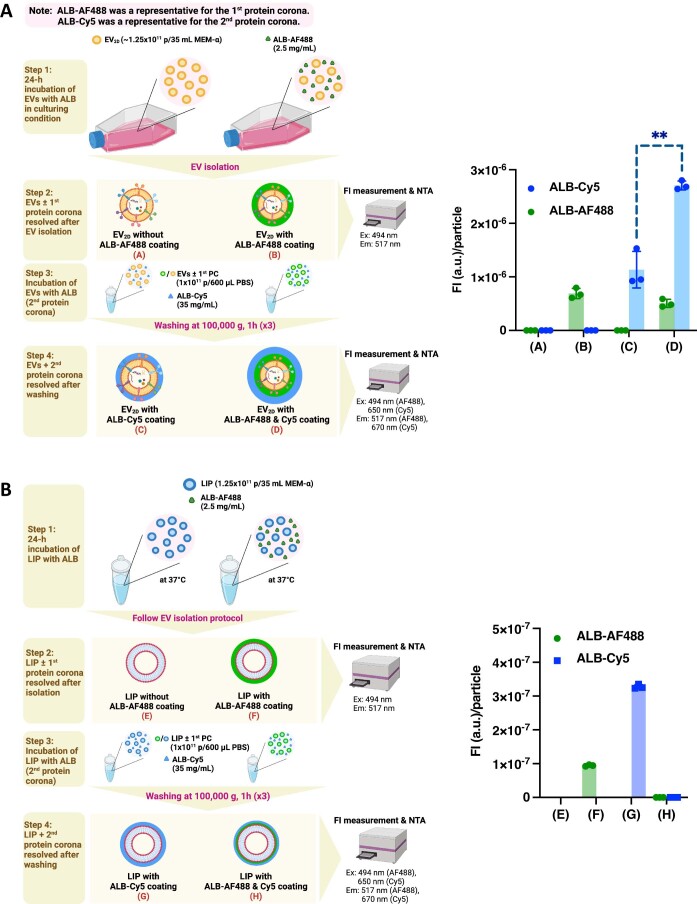
*In vitro* study simulating formation of primary protein corona (1^st^ corona) and secondary protein corona (2^nd^ corona) on EVs and liposomes. (**a**) EV_2D_ and (**b**) Negatively charged liposomes (LIP, as representative of synthetic particles with lipid bilayers) were incubated firstly with albumin labelled with Alexa fluor 488 (ALB-AF488) (1^st^ corona) then with albumin labelled with Cyanide 5 (ALB-Cy5) (2^nd^ corona). Fluorescence was evaluated by a plate reader to determine the degree of albumin adsorption. Results confirmed that EV precoating with ALB (1^st^ corona) induces deposition of more albumin (2^nd^ corona) on EVs (**p = *0.0015*). This is mimicking EVs interaction with blood when injected intravenously. In case of LIP, quenching was observed. Data are presented as mean ± SD (n = 3, biologically independent samples) with two-tailed unpaired t-Test analysis. *The figure was created with BioRender.com*. Source data

To evaluate the first hypothesis, we simulated EV_3D_ culturing supplementation (formation of the first corona) through exposure to Alexa Fluor 488 (AF488)-labelled albumin (ALB-AF488) during the 2D culture, and subsequently incubated the isolated EV_2D_ with Cy5-labelled albumin (ALB-Cy5) to simulate the formation of the second corona. A similar phenomenon to the behaviour of EV_3D_ was observed, that is, isolated EV_2D_ with an albumin-enriched first corona attracted more albumin molecules which formed a second corona layer, thereby proving our hypothesis (Extended Data Fig. 1a). The lack of spillover between two fluorophores was confirmed to ensure the measurement accuracy of EVs with two co-localized fluorescent signals (Supplementary Fig. 21). We visually confirmed that the primary corona is associated with EVs by dual-tracking fluorescence microscopy (Supplementary Fig. 22). A negatively charged liposome (LIP) was included as a synthetic NP control. When implementing dual corona formation, the albumin-enriched corona formed on the LIP was disrupted upon ultracentrifugation, suggesting a weaker association with LIP than observed with EVs (Extended Data Fig. 1b).

**Fig. 6:**
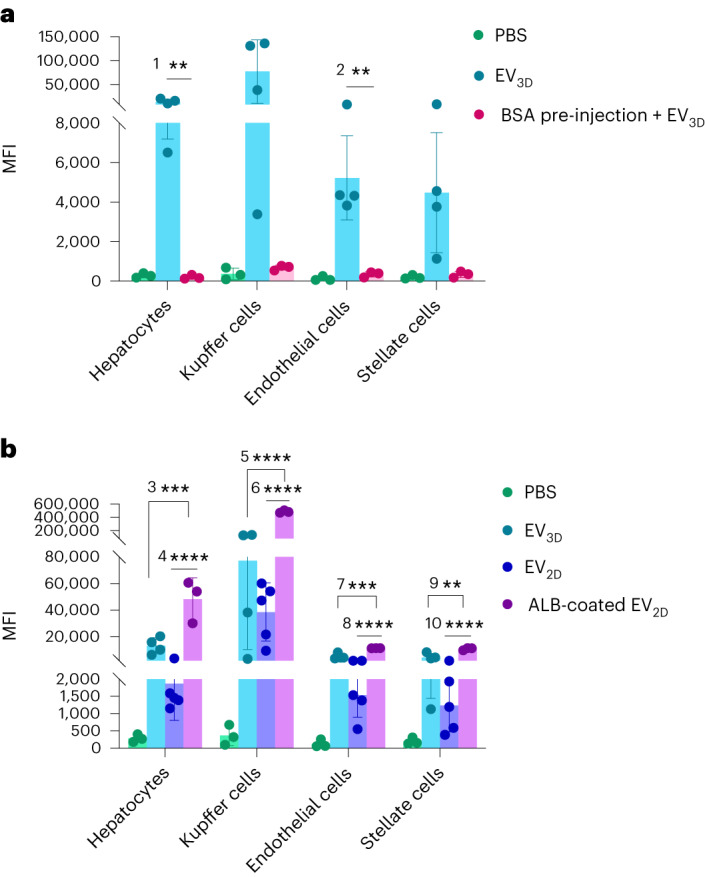
In vivo proof-of-concept studies confirming the effect of albumin receptor saturation or EV coating on liver cell internalization. **a**, In vivo uptake of EV_3D_ in liver cells is reduced/blocked when mice were injected with BSA (5 min pre-EV injection, 10 mg ml^−1^, 100 µl) followed by intravenous injection of DiD-labelled EV_3D_ (2 × 10^11^ particles per mouse, *n* = 3, ^1^**P = 0.0091 and ^2^**P = 0.0063). **b**, In vivo uptake of albumin-coated DiD-labelled EV_2D_ in liver cells after intravenous administration (2 × 10^11^ particles per mouse, *n* = 3, ^3^***P = 0.0004, ^4^****P < 0.0001, ^5^****P < 0.0001, ^6^****P < 0.0001, ^7^***P = 0.0001, ^8^****P < 0.0001, ^9^**P = 0.0018, ^10^****P < 0.0001). This confirms the involvement of albumin and albumin receptor in the cellular internalization of EVs. Data are presented as mean ± s.d. with one-way ANOVA with post hoc Tukey (*P < 0.05, **P < 0.01, ***P < 0.001 and ****P < 0.0001). Source data

To test the second hypothesis, we attempted to block albumin receptors (reviewed in Supplementary Table 9 and Supplementary Fig. 23). Confirming our hypothesis, the uptake of EV_3D_ was reduced in vitro (Supplementary Fig. 24). The in vivo biodistribution and liver uptake was performed by intravenous injection of bovine serum albumin (BSA) followed by DiR or DiD-labelled EV_3D_, respectively. A reduction of EV_3D_ accumulation in the liver at 1 h, 4 h and 24 h and uptake by all liver subpopulations at 24 h could be seen when BSA was coadministered (Supplementary Fig. 25 and Fig. 6a, respectively). In another study, we compared the uptake of DiD-labelled EV_2D_ with and without albumin coating. Albumin-coated EV_2D_ showed the highest uptake by all cell types (Fig. 6b). Surprisingly, preincubation of isolated EV_2D_ with KO media, however, did not lead to cell uptake enhancement (Supplementary Fig. 26), implying that the formation of the first corona at the site of secretion is essential (see Supplementary Fig. 27 for a summary of the in vivo results). Collectively, these studies confirmed the role of albumin receptors in the internalization of albumin-enriched EVs.

## Discussion

It is well established that protein corona formation around synthetic NPs, upon systemic exposure, can be a determining factor for in vivo fate. For EVs, only a few studies have reported the adsorption of proteins acquired from CCM and/or after exposure to serum. Protein corona on THP-1-derived EVs was shown to mediate proinflammatory responses by human-monocyte-derived dendritic cells in vitro^27^. A corona layer of protein secretome was also found on human-platelet-lysate-derived EVs after EV isolation using tangential-flow filtration. This corona exerted regenerative and immunomodulatory properties on the skin-organoid model and T cells in vitro^28^. In line with this study, the presence of corona containing proangiogenic factors secreted from the parent cells, human placental expanded stromal cells, on placental expanded stromal-cell-derived EVs could mediate vascularized skin regeneration in vivo^29^. Our study also confirms that protein corona can form on EVs during the culture process and could contribute to biological changes.

Albumin coating of a range of synthetic nanocarriers has shown beneficial effects, including enhanced lipid membrane interactions, reduced toxicity, augmented receptor-mediated (gp60, gp30, gp18 and SPARC receptors) uptake in tumour cells^30–36^, and prolonged blood circulation^37^. Albumin corona on PHBHHx biopolymer inhibited plasma protein adsorption (that is, free immunoglobulin-G and complement activation fragments, considered as ‘opsonins’), resulting in a lower degree of opsonization^37^. Similarly, albumin-rich corona adsorbed on polymeric molecularly imprinted nanogels reduced non-specific protein binding and increased NP half-life^38^. In our study, we also observed that EVs with albumin-enriched corona adsorbed less immunoglobulin and complement fragments. Albumin has also been employed as drug–NP conjugates for liver non-phagocytic-cell-directed targeting. Naproxen, a conventional anti-inflammatory drug, conjugated with human serum albumin (HSA) was efficiently targeted to LSECs due to the recognition of HSA by scavenger receptors^39^. Correspondingly, liposomes coupled with HSA derivatized with *cis*-aconitic anhydride were mostly taken up by LSECs due to the action of the albumin-scavenger receptor^40^. The uptake of albumin-bound oleate by hepatocytes was also shown to be facilitated by albumin receptors^41^. In line with this, the albumin-dominated corona formed on lipid-like NPs was shown to preferentially bind to gp18 and gp30 expressed on primary hepatocytes, leading to enhanced cellular uptake via macropinocytosis and endocytosis^42^. The hepatic-albumin receptors, therefore, evidently contribute to the uptake of exogenous albumin-bound compounds. In terms of EVs, it was reported recently that EVs decorated with albumin-binding domains could capture more albumin in the circulation, resulting in prolonged half-life^43^. Our results broadly showed hepatic persistence of EV_3D_, whereas EV_2D_ showed susceptibility to renal clearance. The ability of EV_3D_ to evade renal excretion might be explained by the binding affinity of albumin to the receptors responsible for mediating the reabsorption of filtered proteins in a renal proximal tubule, cubilin and megalin, resulting in the albumin–EV complex being rescued from renal clearance^12,13^. Nevertheless, the ability to prolong the blood-circulation time of albumin-enriched corona on EVs should be further investigated in depth.

Although we have confirmed here the contributory effects of corona enriched with albumin, the main component in culture media and systemic environment, the depletion of the other subcomponents, for example, complement proteins, in heat-inactivated serum, which was used to simulate hard corona formation in this study, might affect the profile and amount of adsorbed protein^44,45^. Therefore, the formation of protein corona on EVs in non-heat-activated serum requires additional scrutiny.

## Conclusions

Our delivery complex is proposed as liver cell targeting EVs enabled by albumin preloading during culture processes. Although our study focused on the effect of albumin, the most enriched protein in the corona, on modulation of uptake, a whole series of other studies can follow looking at the function of the individual proteins. Our prototypic model might be applied to develop efficient carrier for EVs from other cell sources. Future work will focus on exploring if these changes can result in improved therapeutic responses in a liver disease model.

## Methods

### Two-dimensional culture of umbilical-cord-derived MSCs

MSCs from umbilical cord (ucMSCs) were obtained from the Anthony Nolan Cord Bank, cut in small pieces and plated in basal medium (that is, αMEM + 1% v/v penicillin–streptomycin) containing 5% (v/v) human platelet lysate at 37 °C, 5% CO_2_. After removal of non-adherent cells and washing with PBS, the media was replenished and the cells were cultured until reaching 70–90% confluency. Trypsinization was then performed for further characterization and cell passaging. ucMSCs were characterized for positivity of CD90, 105, CD106 and CD73, human leukocyte antigen class I, and the lack of expression of CD14, CD31 and CD45. Early passages of 1 × 10^6^ ucMSCs (passages 2–5) were continuously cultured in a filtered 175 cm^2^ flask (Corning) in basal media + 5% (v/v) human platelet lysate. When the cells reached 70%–80% confluency, the media were replaced with basal medium prior to harvesting CCM on the following day for EV isolation and subculturing by a conventional trypsinization protocol.

### Three-dimensional culture of ucMSCs

Aggrewell400 microwell culture plate was used to generate ucMSC spheroids following a previously established protocol^15^. Briefly, 500 µl anti-adherence rinsing solution was added to each well prior to centrifugation at 2,000*g* for 2 min to remove bubbles and incubation for 30 min–2 h at room temperature. The Aggrewell was then washed with 500 µl PBS per well, followed by addition of 500 µl basal medium and centrifugation at 2,000*g* for 2 min to remove bubbles. The basal medium was then replaced with the cell suspension obtained from 2D trypsinization (prepared at a density of 1.2 × 10^5^ cells per 500 µl per well in the basal medium supplemented with 20% v/v KO serum replacement according to the manufacturer’s instructions). The plate was then centrifuged at 200*g* for 5 min for cell aggregation at the bottom of each microwell and kept undisturbed in the incubator. CCM was harvested on day 3 and every 2–3 days for EV isolation. Fresh medium was replenished after medium harvesting to maintain spherical ucMSC culture for 12 days.

### Isolation of EVs

EV isolation was performed as described in detail previously^46^. Ultraclear polycarbonate ultracentrifuge tubes (catalogue number 355631, Beckman Coulter) were filled with 22.5 ml filtered CCM (prepared by filtering CCM using a 0.22 µm syringe filter). Then, 3 ml 25% w/w sucrose solution (prepared in D_2_O) was layered slowly below the CCM using a glass Pasteur pipette. Centrifugation using a swing-out rotor (SW32 Ti, Beckman Coulter) was performed at 100,000*g* for 1.5 h at 4 °C. The sucrose solution was then collected (2 ml per tube) and subjected to a washing step for EV purification by adding to prefilled polycarbonate ultracentrifuge tubes (catalogue number 355618; Beckman Coulter) with 20 ml filtered PBS prior to ultracentrifugation at 100,000*g* for 1.5 h at 4 °C using a fixed-angle rotor (70 Ti, Beckman Coulter). Supernatant was discarded and the pellet of EVs obtained was resuspended in filtered PBS. EVs were kept at 4 °C for 1 week storage and −80 °C for long-term storage.

### Detection of EV markers by dot-blot

The analysis was performed following a previously published protocol^15^. First, 50 µl EVs at a concentration of 5 × 10^10^ particles per ml were spotted on a nitrocellulose membrane (one membrane per marker) (Bio-Rad). The membrane was dried by nitrogen gas prior to the blocking step using blocking buffer (that is, 3% skim milk prepared in Tris-buffered saline with 0.1% Tween 20 (TBS-T)) for 1 h at room temperature. Primary antibodies (CD9, CD63, CD81 and TSG101) were individually added in fresh blocking buffer followed by incubation overnight at 4 °C (1:1,000 dilution). The membrane was then washed three times in TBS-T, 5 min each wash. Horseradish-peroxidase-conjugated secondary antibody was then added to the fresh blocking buffer prior to further incubation for 1 h at room temperature (1:20,000 for anti-mouse and 1:1,000 for anti-rabbit). The membrane was washed as previously mentioned, and the signals were developed by substrate addition (SuperSignal West Femto Maximum Sensitivity Substrate), followed by imaging using the Gel Doc system (Bio-Rad) and analysis by Image Lab software (Bio-Rad).

### Protein assay for determining the protein concentration of isolated EVs

Protein concentrations were determined by microBCA assay in a 96-well plate following the manufacturer’s instructions adapted for EVs. Briefly, EV samples (minimum concentration, 5 × 10^10^ particles per ml) were diluted 1:1 in PBS. MicroBCA reagent mix (prepared according to the manufacturer’s instructions) was added to duplicate 40 µl diluted samples (50 µl per well), followed by incubating at 37 °C for 1 h. The measurement was compared against serially diluted BSA as standard (prepared in duplicates). The absorbance was read at 562 nm using a FLUOStar Omega plate reader (BMG LabTech). MARS v.2.40 software (BMG LabTech) was used for analysis by extrapolating the values from the standard curve using a third-order polynomial equation, with *r*^2^ > 0.999 for each assay.

### NTA

The size and concentration of EVs was measured by NTA using a Nanosight LM10 (Malvern Instruments) equipped with a 488 nm laser. The camera level was automatically adjusted, and the analysis detection threshold was set at 3–4. EV samples were diluted in filtered PBS to obtain optimal concentrations (20–80 particles per frame). Four video recordings with a duration of 40 s were carried out for each EV preparation. Nanosight NTA 3.2 software (Malvern Instruments) was used to analyse the recorded video.

### Zeta-potential

The dynamic electrophoretic mobility of EVs and HC-EVs was measured with a Malvern Zetasizer Nano ZS and Zetasizer v.7.12 software (Malvern Instruments). Prior to the measurements, EV samples (minimum concentration, 1 × 10^11^ particles per ml) were diluted 1:50 in deionized water. Measurements were carried out at 25 °C for each experimental triplicate.

### Protein corona preparation

Protein corona coating was performed following a published protocol^47^ with modifications. To remove EVs and aggregated protein serum, FBS was subjected to ultracentrifugation at 100,000*g* for 18 h at 4 °C. Supernatant was collected and filtered through 0.22 µm filters (EV-D FBS). EVs (∼7 × 10^11^ particles, equivalent to 0.05 m^2^) were prepared in a total volume of 300 µl sterile PBS supplemented with 1% penicillin–streptomycin solution (sPBS-PS) in 1.5 ml Eppendorf tubes prior to incubating with 300 µl EV-D FBS for 1 h at 37 °C, 300 r.p.m. Upon incubation, the mixture was transferred to an ultracentrifuge tube (catalogue number 343778, Beckman Coulter). The samples were ultracentrifuged at 100,000*g* for for 1 h at 4 °C. Supernatant was carefully removed without disturbing the pellet. The pellet was further washed with 1 ml sPBS-PS twice, following the above-mentioned condition. In corona desorption studies, upon the final wash, the pellet (HC-EVs) was dispersed in freshly prepared 100 µl 2% w/v SDS, 62.5 mM Tris–HCl solution, followed by incubation at 95 °C for 5 min to desorb the proteins constituting the HC. The HC was then separated from EVs by ultracentrifugation as above. The supernatant containing HC was collected and subjected to protein determination by microBCA assay. HC solutions were stored at −80 °C before further analysis.

### SDS–PAGE

EVs, HC-EVs, EV-D FBS, KO and HC (15 µg) samples were mixed with LDS sample buffer and RIPA buffer (mixed with 1:100 protease inhibitor cocktail) and incubated for 10 min at 70 °C. The samples were then applied on a NuPAGE 4–12% Bis–Tris protein gel and run for 30 min at 100 mV and a further 1 h at 150 mV. For silver staining, the gel was fixed in 100 ml fixing solution (50% methanol, 10% acetic acid, 50 µl formaldehyde) overnight, followed by washing with 50% ethanol three times. The gel was then sensitized by hypo-solution (0.02% w/v sodium thiosulfate) for 1 min, followed by impregnating the gel with 0.2% w/v silver nitrate for 30 min. The gel was then washed with deionized water three times and subjected to band development using 100 ml developing solution (6 g sodium carbonate, 2 ml hypo-solution and 50 µl formaldehyde). Once developed, the reaction was stopped by 5% v/v acetic acid. A digital photograph of the gel was taken against a white background.

### Digestion of the protein corona for mass spectrometry analysis

Proteomic analysis of FBS proteins, EVs and their HC by LC–MS was conducted using the method described by Schottler et al.^47^. Briefly, SDS was removed from the samples by Pierce detergent removal columns, and 25 µg of each protein sample was precipitated by ProteoExtract protein precipitation kit following the manufacturer’s manual. The resulting protein pellets were resuspended in 0.1% RapiGest SF in 50 mM ammonium bicarbonate and incubated for 15 min at 80 °C. Dithiothreitol (final concentration, 5 mM) was added to reduce the proteins, and the mixture was incubated at 56 °C for 45 min. Iodoacetamide (final concentration, 15 mM) was then added, and the mixture further incubated in the dark for 1 h at room temperature. The protein samples were digested for 18 h at 37 °C by trypsin with an enzyme:protein ratio of 1:50 (w/w). The reaction was stopped by the addition of 2 μl hydrochloric acid. The digested peptides were diluted with 0.1% v/v formic acid in UPLC-MS grade water and spiked with 50 fmol μl^−1^ Hi3 *Escherichia coli* standard for absolute quantification.

### In vitro cellular uptake of EVs and HC-EVs

EV_2D_ and EV_3D_ were labelled as per our published protocol^48^. Concentration in particles per ml were obtained by NTA as mentioned above. The fluorescence intensity of freshly prepared EVs (100 µl) was measured using a FLUOStar Optima plate reader (BMG Labtech), with excitation and emission wavelengths of 485 nm and 520 nm, respectively. When there is a difference in labelling efficiencies between the batches or EV types, samples were mixed with non-labelled EVs so that fluorescence intensity and particles numbers are comparable for the different samples. Phagocytic cells, that is, J774 and human-monocyte-derived macrophages, and non-phagocytic cells, that is, HepG2, were seeded at a density of 1.5 × 10^5^ cells per well in 24-well plates (Corning). After 24 h, the media were changed to serum-free media, and the cells were treated with labelled EV_2D_, HC-EV_2D_, EV_3D_ and HC-EV_3D_ of comparable fluorescence unit values and a dose of 2 × 10^9^ particles and incubated for 1 h, 4 h and 24 h. To evaluate the contribution of albumin-binding receptor in mediating the EV internalization, HepG2 cells were treated with BSA (12.5 mg ml^−1^), followed by the addition of DiD-labelled EV_3D_, for 24 h. Flow cytometry was performed on a BD FACSCalibur using BD FACStation v.6.0 software (BD Biosciences) on the detached cells by trypsin–EDTA 0.05%, washed and resuspended in 200 µl PBS. All samples were analysed using FlowJo v.10.7.2 (TreeStar/BD Bioscience). Cells were gated by their forward and side scatter. The uptake of EVs was evaluated by the MFI of Alexa Fluor 488. Fold change in MFI was calculated with respect to the relative MFI increase over untreated controls.

### Animals

All animal experiments were performed in compliance with the UK Animals (Scientific Procedures) Act 1986 and the UK Home Office Code of Practice for the Housing and Care of Animals Used in Scientific Procedures (Home Office 1989). In vivo experimentation adhered to the project license approved by the King’s College London animal welfare and ethical review body (AWERB) and the UK Home Office (PBE6EB195 and PP8950634). Animal research and veterinary care was performed at Franklin-Wilkins Building, King’s College London under the protocol approved for this study by a Named Training and Competency Officer (Julie Keeble) and a Named Animal Care and Welfare Officer (Jayne Morgan). Female CD-1 mice (~25–35 g, 5 weeks old) were obtained from Charles River for in vivo biodistribution study. Male and female C57BL/6 mice (18–25 g, 6–8 weeks old) were obtained from Charles River for in vivo cellular uptake study. All mice were housed in a 12 h light/12 h dark cycle with the temperature maintained between 65 and 75 °F (∼18–23 °C) and ∼50% humidity. Both sexes were used in the study, complying with the recently published recommendations by the UK Medical Research Council for conducting research on animals.

### In vivo biodistribution of EVs by fluorescence optical imaging

Filtered CCM was incubated with 1 µM fluorescent lipophilic tracer DiR (1,1-dioctadecyl-3,3,3,3-tetramethylindotricarbocyanine iodide) at room temperature for 1 h with agitation prior to EV isolation by sucrose cushion plus ultracentrifugation as described above to remove unbound dye. The experiment was performed in CD1 mice, randomly divided into groups. Freshly purified DiR-labelled EV_2D_ and EV_3D_ (2 × 10^11^ particles in 200 µl) were injected intravenously via the tail vein. Control mice were injected with PBS only or PBS containing DiR. Isoflurane-sedated live mice were imaged using the IVIS Lumina III system (excitation, 740 nm; emission, 840 nm) at 1, 4 and 24 h following intravenous injection prior to killing the animals. Major organs (brain, heart, lung, liver, stomach, spleen, kidney and intestine) were harvested for ex vivo fluorescence imaging. The fluorescence intensity in each organ (total radiant efficiency) was obtained using Living Image v.4.7.3 Software (PerkinElmer) to determine the organ biodistribution of DiR-labelled EV_2D_ and EV_3D_ by drawing the regions of interest (ROIs) of each organ. The values were then normalized to organ weights (total radiant efficiency per g). For the renal clearance study, upon injection with DiR-labelled EVs, mice were individually housed in a standard circular metabolic cage (Nalgene Nunc) for 24 h, and urine was collected into a Nalgene tube at the bottom of a funnel system for further fluorescence intensity determination using an IVIS Lumina III system and Living Image v.4.7.3 Software (PerkinElmer) by drawing ROIs covering a urine container to obtain total radiant efficiency per urine sample.

### In vivo cellular uptake of EVs in liver subpopulations

EV_2D_ and EV_3D_ were labelled with DiD (DiIC18(5); 1,1′-dioctadecyl-3,3,3′,3′-tetramethylindodicarbocyanine, 4-chloro-benzenesulfonate salt) using the same procedure as for DiR labelling in the in vivo studies. The experiment was performed in C57BL/6 mice, randomly divided into groups. Freshly purified DiD-labelled EV_2D_ and EV_3D_ (2 × 10^11^ particles in 200 µl) were intravenously injected via the tail vein. Control mice were injected with PBS only or PBS containing DiD. For the albumin receptor blocking study, 100 µl BSA diluted in PBS (10 mg ml^−1^, dosage selected based on the literature^49–52^) was injected 5 min prior to EV_3D_ injection (2 × 10^11^ particles in 100 µl). For the study of albumin-coated EV internalization, to prepare albumin-coated, DiD-labelled EV_2D_, 2D ucMSCs (70–80% confluency) were cultured in basal medium supplemented with 2.5 mg ml^−1^ BSA, followed by medium collection after 24 h of culture, DiD labelling and EV isolation as previously mentioned. Albumin-coated DiD-labelled EV_2D_ were then intravenously injected (2 × 10^11^ particles in 200 µl). Twenty-four hours later, mice were anaesthetized with phenobarbital and subjected to skin dissection on the ventral midline to open the peritoneal cavity for liver perfusion following the previously published protocol with modifications^53^. Mice were perfused with 30 ml HBSS–EGTA warmed at 41 °C through the inferior vena cava using a peristaltic pump (SciQ 300, Watson Marlow) and a 27G × 0.38″ × 12″ winged infusion set (BD Valu-Set) at a speed of 15 r.p.m. The hepatic portal vein was cut after the liver became swollen and discoloured, after which the speed was adjusted to 20 r.p.m. Mice were then perfused with 25 ml collagenase-containing HBSS–CaCl_2_ (concentration, 1 mg ml^−1^) warmed at 41 °C, at a speed of 15 r.p.m. The liver was transferred to a Petri dish containing cold HBSS–CaCl_2_ buffer. Hepatic cells were released by gentle breaking of Glisson capsule and shaking the liver. Upon obtaining homogeneous cell suspension, cells were filtered through a 70 µm strainer (Corning). Hepatocytes were separated by centrifugation for 3 min at 50*g* at 4 °C with low brake, three spins. After each spin, the pellet was resuspended in 30 ml cold HBSS–CaCl_2_, and the supernatant was collected for further NPC fractionation. The NPC fraction was pelleted at 650*g* at 4 °C and subjected to incubation with RBC lysis buffer for 5 min on ice, followed by addition of 20 ml PBS to stop the reaction and further centrifugation at 650*g* at 4 °C to pellet the purified NPC fraction. The identification of each subpopulation was performed based on the marker expression using fluorescently labelled antibodies for analysis by flow cytometry (BD FACSCelesta, operated by BD FACSDiva v.9.2 software, BD Biosciences) and FlowJo v.10.7.2 software (TreeStar/BD Bioscience). Hepatocytes were stained with anti-mouse ASGPR1 antibody (1:200)^54–56^ and anti-human CD9 via intracellular staining using 0.1% Triton-X 100 in PBS. Kupffer cells were stained with anti-mouse CD45, F4-80 and CD11b (1:200, each)^57–59^. Endothelial cells were stained with anti-mouse CD45 (1:200), CD31 (1:200) and CD146 (1:100)^60–62^. Stellate cells were stained with anti-mouse CD45 (1:200), GFAP (1:50) and detected using a 450/50 filter and a 405 nm violet laser^63–65^. All fractions were stained with Zombie Aqua to determine the viability of the cells. Staining was performed for 30 min at room temperature. The frequency of parent cells taking up EVs was defined by gating the DiD-positive cell population against the control (PBS) group. The MFIs of the DiD signal expressed by each cell type were used to evaluate the uptake amount of EVs.

### Statistical analysis

Statistical analyses of the data were performed using Prism 9.4.1 (GraphPad Software) by using one-way analysis of variance (ANOVA) with Tukey post hoc test for all *P* values (*P < 0.05, **P < 0.01, ***P < 0.001, ****P < 0.0001; *P* > 0.05 was non-significant). All results are expressed as mean ± s.d. Data distribution was assumed to be normal but this was not formally tested. No statistical methods were used to predetermine sample sizes, but the sample sizes were chosen based on previous experiments similarly performed by our group with proven statistically significant effects^10^. All graphs were made in Prism 9.4.1. MATLAB 9.11 was used to generate heatmaps of data. Data collection and analysis were not performed blind to the conditions of the experiments. All data points were included for analyses.

### Reporting summary

Further information on research design is available in the Nature Portfolio Reporting Summary linked to this article.

## Online content

Any methods, additional references, Nature Portfolio reporting summaries, source data, extended data, supplementary information, acknowledgements, peer review information; details of author contributions and competing interests; and statements of data and code availability are available at 10.1038/s41565-023-01585-y.

### Supplementary information

Supplementary Information Supplementary Materials, Methods, Results, Figs. 1–27, Tables 1–9 and References.

Reporting Summary

Supplementary Data Source data for Supplementary Figs. 4, 9, 10, 15, 17, 18, 20, 24, 26.

### Source data

Source Data Fig. 1 Numerical source data and uncropped dot-blots, respectively.

Source Data Fig. 2 Numerical source data and uncropped gels, respectively.

Source Data Fig. 3 Numerical source data.

Source Data Fig. 4 Numerical source data.

Source Data Fig. 5 Numerical source data.

Source Data Fig. 6 Numerical source data.

Source Data Extended Data Fig./Table 1 Numerical source data.

## Data Availability

UniProtKB/SwissProt *Homo sapiens* and *Bos taurus fasta* databases (Proteome ID: UP000005640 and UP000009136, respectively) were used in this study. The authors declare that all data supporting the findings of this study are available in the provided Source Data and the Supplementary Information. Additional data are also available from the corresponding author upon reasonable request. Source data are provided with this paper.
