# Supplementary material for: Cellular uptake and in vivo distribution of mesenchymal-stem-cell-derived extracellular vesicles are protein corona dependent
Source: Nat Nanotechnol. 2024 Feb 16;19(6):846–55. doi: 10.1038/s41565-023-01585-y (PMC11186763; doi:10.1038/s41565-023-01585-y)
Supplement: Supplementary file 1 — Supplementary Materials, Methods, Results, Figs. 1–27, Tables 1–9 and References. [file 41565_2023_1585_MOESM1_ESM.pdf]

nature nanotechnology

Article

https://doi.org/10.1038/s41565-023-01585-y

Cellular uptake and in vivo distribution of mesenchymal-stem-cell-derived extracellular vesicles are protein corona dependent

In the format provided by the authors and unedited

Table of Contents

Supplementary Materials 3

Supplementary Methods 5
- Preparation of bovine serum albumin labelled with fluorescence dye 5
- Liquid chromatography mass spectrometry (LC-MS) analysis 5
- Data processing and protein identification 5
- Gene ontology (GO) enrichment analysis 6
- Transmission electron microscopy 6
- The effect of cell architecture on the protein identity of EVs. 6
- Fluorescence microscopy 7
- Albumin receptors blocking studies *in vitro.* 7

Supplementary Results 8
- The effect of cell architecture on the protein identity of EVs. 8
- Labelling efficiency of Alexa488 and DiD-labelled EVs 8

Supplementary Figures 9
- Figure S1 9
- Figure S2 10
- Figure S3 11
- Figure S4 12
- Figure S5 13
- Figure S6 14
- Figure S7 15
- Figure S8 16
- Figure S9 17
- Figure S10 18
- Figure S11 19
- Figure S12 20
- Figure S13 21
- Figure S14 22
- Figure S15 23
- Figure S16 24
- Figure S17 25
- Figure S18 26
- Figure S19 27
- Figure S20 28
- Figure S21 29
- Figure S22 30
- Figure S23: 31
- Figure S24 32
- Figure S25 33
- Figure S26 34
- Figure S27 35

Supplementary Tables 36
- Table S1 36
- Table S2 37
- Table S3 38
- Table S4. 39

Table S5 41
Table S6 42
Table S7 43
Table S8 44
Table S9 45

Supplementary References.. 46

Supplementary Materials

Sterile heat-inactivated fetal bovine serum (FBS) was purchased from First Link (UK). Human platelet lysate (hPL) was purchased from Sexton Biotechnologies (USA). PBS pH 7.4 10X, MEM-alpha, penicillin/Streptomycin, GlutaMax™ 100X, Trypsin EDTA 0.05%, KnockOut™ (KO) serum replacement, SuperSignal™ West Femto Maximum Sensitivity Substrate, Pierce™ Bovine Serum Albumin Standard Ampules, 2 mg/mL, Micro BCA™ kit, Pierce detergent removal columns, NuPAGE™ Lithium Dodecyl Sulfate (LDS) sample buffer, and NuPAGE™ 4-12% Bis-Tris protein gel were purchased from Thermo Fisher Scientific (UK). Complete™ Protease Inhibitor Cocktail was purchased from Roche (Switzerland). Aggrewell™400 microwell culture plate and anti-adherence rinsing solution were purchased from STEMCELL Technologies (France). Sucrose and acetic acid were purchased from Fisher Scientific (UK). Deuterium oxide, glutaraldehyde, Tween® 20, sodium cacodylate, sodium dodecyl sulphate (SDS), RIPA buffer, and 16% paraformaldehyde were purchased from Sigma Aldrich (UK). Nitro-cellulose membrane was purchased from Bio-Rad (UK). Skim milk powder and bovine serum albumin were purchased from Fluka Analytical (Germany). Purified anti-human CD9 (clone: HI9a), CD81 (clone: 5A6), RBC lysis buffer 10X, anti-mouse CD45 (PE and PerCP, clone 30-F11), F4/80 (FITC, clone BM8), CD11b (PerCP, clone M1/70), CD31 (PE, clone 390), and Zombie Aqua™ were purchased from Biolegend (UK). Purified anti-human CD63 (clone: EPR5702) was purchased from Abcam (UK). Anti-mouse and rabbit IgG HRP-linked were purchased from Cell Signalling Technology (USA). Anti TSG101 (polyclonal, 14497-1-AP) was purchased from Proteintech (USA). Silver nitrate was purchased from VWR international (UK). ProteoExtract® protein precipitation kit was purchased from Calbiochem® (USA). RapiGest™ SF and Hi3 Ecoli Standard were purchased from Waters Corporation (USA). DiR (1,1-dioctadecyl-3,3,3,3-tetramethylindotricarbocyanine iodide), DiD (DiIC18(5); 1,1′-dioctadecyl-3,3,3′,3′-tetramethylindodicarbocyanine, 4-chloro-benzenesulfonate salt), and Alexa Fluor™ 488 azide were purchased from Invitrogen, Life Technologies (UK). Isoflurane (IsoFlo®) for anaesthesia was purchased from Abbott Laboratories (UK). Euthatal (pentobarbital sodium) was purchased from Merial Animal Health Ltd (UK). Hanks' Balanced Salt Solution (HBSS) 10X and Anti-ASGPR1/Asialoglycoprotein Receptor 1/ASGR1 Antibody (clone 8D7) Alexa Fluor® 647 were purchased from Santa Cruz Biotechnology (USA). Collagenase type IV was purchased from Worthington Biochemical Corporation (USA). Anti-human/mouse/rat GFAP, REAfinity™ (clone REA335) and anti-mouse CD146-FITC (clone ME9F1) were purchased from Miltenyi Biotec (Germany). Mouse SPARC Alexa Fluor 647-conjugated antibody (clone 124413) was purchased from, R&D systems (UK). DBCO-NHS ester, Alexa-Fluor™488 NHS ester, Cyanin5 (Cy5) NHS ester were purchased from Lumiprobe (Germany). NAP™5 column was purchased from Cytiva (USA). J774A.1 (BALB/cN mouse macrophage, ATCC® TIB-67TM) and HepG2

(human hepatocellular carcinoma, ATCC® HB-8065 were obtained from ATCC®. Human monocyte-derived macrophages were isolated from human peripheral blood mononuclear cell (PBMCs) by Pan Monocyte Isolation Kit II (Miltenyi Biotec, Germany). The PBMCs were kindly provided by Prof Francesco Dazzi (stored under the Human Tissue Authority (UK) license no.11023).

Supplementary Methods

Preparation of bovine serum albumin labelled with fluorescence dye

Bovine serum albumin (BSA) was conjugated with either Alexa-fluor™ 488 NHS ester or Cyanine-5 NHS ester following the manufacturer's protocol. Briefly, 8 molar excess of NHS ester were reacted with BSA in 9/10 reaction volume of 0.1 M sodium bicarbonate and incubated at RT for 4 h. BSA was purified from the conjugated dye using NAP™5 column and further used for *in vitro* studies to simulate corona formation on EVs. Standard curves of Alexa-fluor 488 spiked with Cy5 were firstly prepared to validate the fluorescence intensity measurement of the samples co-localised with two fluorophores. Dye-conjugated BSA was further used to fabricate ALB-coated EVs.

Liquid chromatography mass spectrometry (LC-MS) analysis

LC-MS analysis was performed using a nanoACQUITY ultra high-performance liquid chromatography (UPLC) system coupled with a Synapt G2-Si mass spectrometer (Waters Corporation, USA). Digested peptides were separated on the nanoACQUITY system equipped with a C18 analytical reversed-phase column (1.7 µm, 75 µm × 150 mm) and a C18 nanoACQUITY trap column (5 µm, 180 µm × 20 mm) with a mobile phase A consisting of 0.1% v/v formic acid in water and a mobile phase B consisting of acetonitrile with 0.1% (v/v) formic acid at a flow rate of 0.3 µL min^{-1}, using a gradient of 2–40% mobile phase B over 65 min. One hundred fifty fmol µL−1 Glu-Fibrinopeptide was infused at a flow rate of 0.5 µL min^{-1} as a reference compound.

Data processing and protein identification

Data-independent acquisition (MSE) experiments were performed on the Synapt G2-Si carried out in resolution mode. Electrospray Ionization was performed in positive ion mode with NanoLockSpray source. Data was acquired in a mass to charge range of m/z 50–2000 Da with a scan time of 1 s, ramped trap collision energy from 20 to 40 V with a total acquisition time of 90 min. All samples were analysed in three replicates. Data acquisition and processing were performed using MassLynx 4.1. Progenesis® QI for Proteomics software Version 2.0 was used to process data and to identify the proteins searched against a reviewed human and bovine database (UniProt). Sequence information of Hi3 Ecoli standard was added to the database to conduct the absolute quantification. Noise reduction thresholds were set for low energy, high energy, and peptide intensity to 120, 25, and 750 counts respectively. The following parameters were set for the peptide and protein identification: Maximum protein mass 600 kDa, one missed cleavage, fixed carbamidomethyl modification for cysteine, variable oxidation for methionine and false discovery rate of 4% for proteins. At least two assigned peptides and five

assigned fragments are required for the protein identification. Quantitative identification of each identified protein in femtomole was provided based on the TOP3/Hi-3 method. Relative abundance percentage (RPA) for each protein could be obtained by proportional comparison between proteins in the same run over the full quantification range.

Gene ontology (GO) enrichment analysis

The UniProt ID of the proteins detected by LC-MS were converted to gene names to obtain the dataset of EV genes and HC genes which were further subjected to gene ontology (GO) enrichment analyses using FunRich software v.3.1.3 (http://www.funrich.org). The in-built hypergeometric statistic test was used to provide evidence for gene classification based on various aspects (e.g., cellular component and biological process) to identify significantly overrepresented than would be expected by chance. The analysis was performed against in-built human and non-human mammal database, for EV and HC datasets, respectively. To further narrow down, GO analysis on cellular component was firstly performed, followed by biological processes implicating clearance. The significant enriched genes were defined by the significance threshold of Bonferroni corrected p-value < 0.05 and underwent Principal Component Analysis (PCA) by inputting the protein relative abundance percentage into the software (Minitab statistical software V20). Score plots, loading plots, and scree plots were used to evaluate the characteristic of each sample type.

Transmission electron microscopy

Transmission electron microscopy (TEM) analysis was conducted following the previously published protocol with slight modification [1]. Briefly, EVs or HC-EVs were fixed in 4% paraformaldehyde in equal volume (final concentration of 2% paraformaldehyde) for 30 min. EV or HC-EV samples were immediately placed and allowed to adsorb on top of a carbon-coated 300-mesh copper grid for 15 min at RT. Grids were washed with PBS and fixed in 1% glutaraldehyde prepared in 0.1M sodium cacodylate for 5 min at RT. The grids were then washed with PBS, blotted dry with filter paper, and negatively stained with UA-Zero non-radioactive EM Stain, followed by washing and drying. TEM visualizations were performed using a FEI Tecnai 12 G2 spirit with an 11-megapixel Olympus sis side mount morada camera at 80 kV.

The effect of cell architecture on the protein identity of EVs.

MSCs were cultured in 2D and 3D conditions as previously mentioned. The media were changed to basal media w/ and w/o KnockOut™ SR supplementation 24 hours prior to conditioned culture media (CCM) harvest. The collected CCM was subjected to EV isolation. The retrieved pellets were suspended

in 50 µL RIPA buffer, followed by protein measurement by microBCA, SDS-PAGE, and silver staining as previously mentioned.

Fluorescence microscopy

Alexa Flour 488-labelled albumin (ALB-AF488) was added to 2D MSC cultured in the supplement-free CCM (2.5 mg/mL) to obtain EV_{2D} covered with ALB-AF488 enriched 1st corona. After 24-h incubation, CCM was collected and incubated with DiI (1µM) to label EVs. DiI-labelled EVs with ALB-AF488 enriched 1st PC, retrieved from the EV isolation, were then incubated with HepG2 cells for 24 h.

To obtain EVs with 1st and 2nd corona, EVs with ALB-AF488 enriched 1st PC were incubated with Cy5-labelled albumin (ALB-Cy5) (35 mg/mL) for 1 h, followed by EV isolation to obtain EV_{2D} with ALB-enriched 1st & 2nd corona which were then incubated with HepG2 cells for 24 h.

To visualise co-localisation of EVs with primary and secondary corona, HepG2 cells (8×10^4) were seeded on coverslips in a 24-well plate for 24 h. Cells were treated with 1×10^9 EVs (DiI-labelled EVs, DiI-labelled EVs incubated with ALB-AF488 or EVs incubated with ALB-AF488 (1st corona) and ALB-Cy5 (2nd corona) for 24 h. Cells were PBS-washed followed by 4% paraformaldehyde (PFA) fixation for 15 min RT. Images were acquired using a Nikon epifluorescence microscope TS2R and analysed using NIS-Elements BR 5.30.04 software (Nikon Instruments, USA).

Albumin receptors blocking studies *in vitro.*

At first, fluorescence intensity of fluorescently labelled BSA suspended in FBS-free DMEM at the concentration of 2.5, 5, 7.5, 10, and 12.5 mg/mL were measured by FLUO star OPTIMA plate reader (BMG Labtech, USA) to obtain '$FI_{initial}$' before addition to HepG2 cells seeded in 24-well plate (1.5×10^4). This was done to establish BSA concentrations sufficient to block albumin receptors. After 24-h incubation, CCM were collected to measure 'FI_{after}' of the fluorescently labelled albumin. The value of FI_{after} divided by $FI_{initial}$ closer to 1 indicates less albumin taken up by cells from the media (**Figure S24A**). The BSA concentration of 12.5 mg/mL was chosen for blocking studies as it is deemed to be the saturation concentration of albumin receptors. After 24-h incubation, cells were trypsinised for flow cytometry analysis to determine the cellular uptake of DiD-labelled EV_{3D} with and without excess BSA incubation.

Supplementary Results

The effect of cell architecture on the protein identity of EVs.

To exclude that this is a function of the cells' architecture (spheroids vs monolayers), we cultured MSC for 24 h in 2D and 3D conditions in the basal media w/ and w/o KO. The results indicated that the supplemental protein in the culture media was mainly responsible for the difference in the EVs' protein profile (**Figure S5**).

Labelling efficiency of Alexa488 and DiD-labelled EVs

It is hypothesised that EV_{3D} will have higher surface labelling efficiency compared to EV_{2D} due to the presence of excess amine groups in the primary corona [2]. Surface labelling of EVs by AF488 azide using DBCO-NHS linker resulted in dramatically higher fluorescence intensity for EV_{3D} than EV_{2D} **(Figure S9A)**. Membrane labelling, however, using the lipophilic dye DiD, resulted in comparable fluorescence intensities for both types **(Figure S9B)** confirming this hypothesis. This is also in line with the previous study done by Smyth *et al.*, indicating that the functionalisation of EVs' surface did not affect the incorporation of DiD into the EV membrane [2].

Supplementary Figures

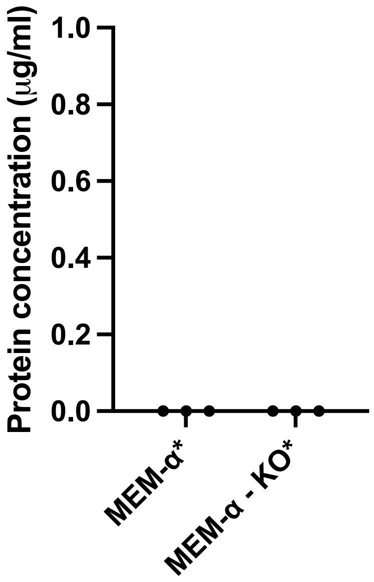

Figure S1: Validation of EV isolation protocol. The protocol was validated by including the un-conditioned media for 2D culture (MEM-α, n=3) and 3D culture (MEM-α containing KO, n=3) to confirm that EVs could be isolated without contamination from the proteins present in the media. *The values '0' shown in the graph refers to the protein concentration which was below than standard curve range. Therefore, the actual values could not be derived.

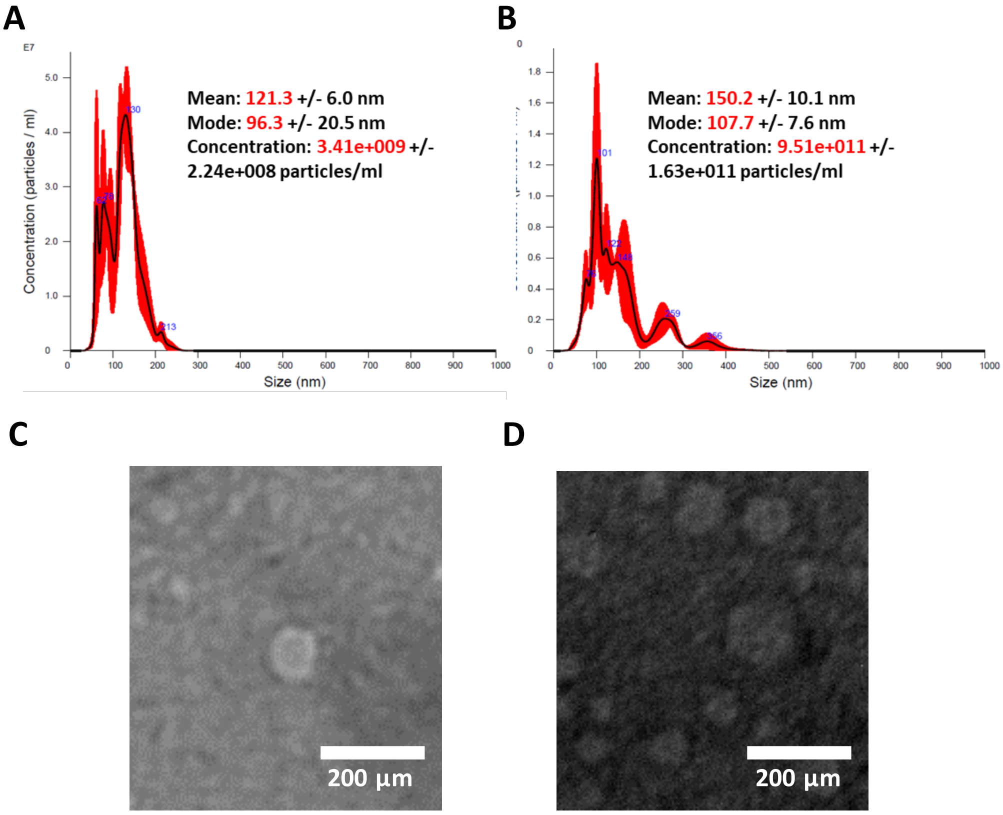

Figure S2: Characterisation of EVs derived from MSCs. (A) & (B) Representative nanoparticle tracking analysis (NTA) of EV_{2D} and EV_{3D}, respectively. Data are presented as mean ± standard error **(C) & (D)** Negative transmission electron microscopy (TEM) of EV_{2D} and EV_{3D}, respectively (n=1).

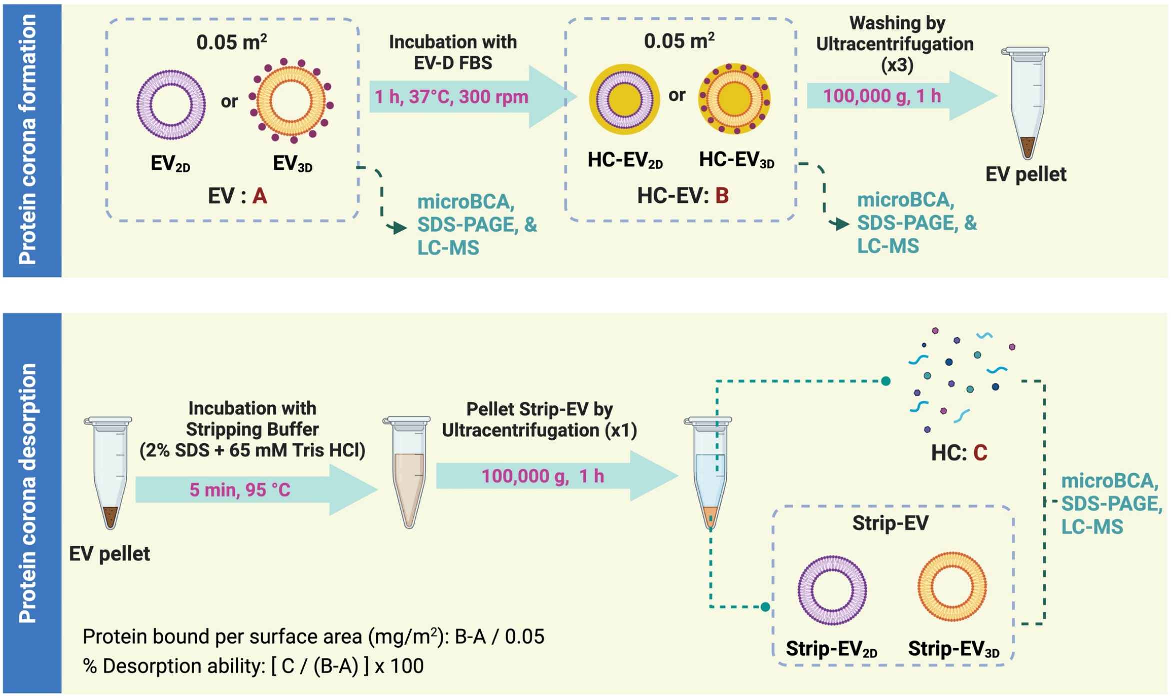

Figure S3: Schematic illustration of hard protein corona (HC) studies. EV_{2D} and EV_{3D} equivalent to 0.05 m^2 were incubated with EV-depleted FBS (EV-D FBS) to allow protein corona formation on the EV surface. HC-EV complexes were separated from unbound proteins by ultracentrifugation, washed then subjected to desorption for the detachment of the HC which was collected by ultracentrifugation. Qualitative and quantitative analyses of HC-EVs and HC were carried out. *The figure was created with BioRender.com.*

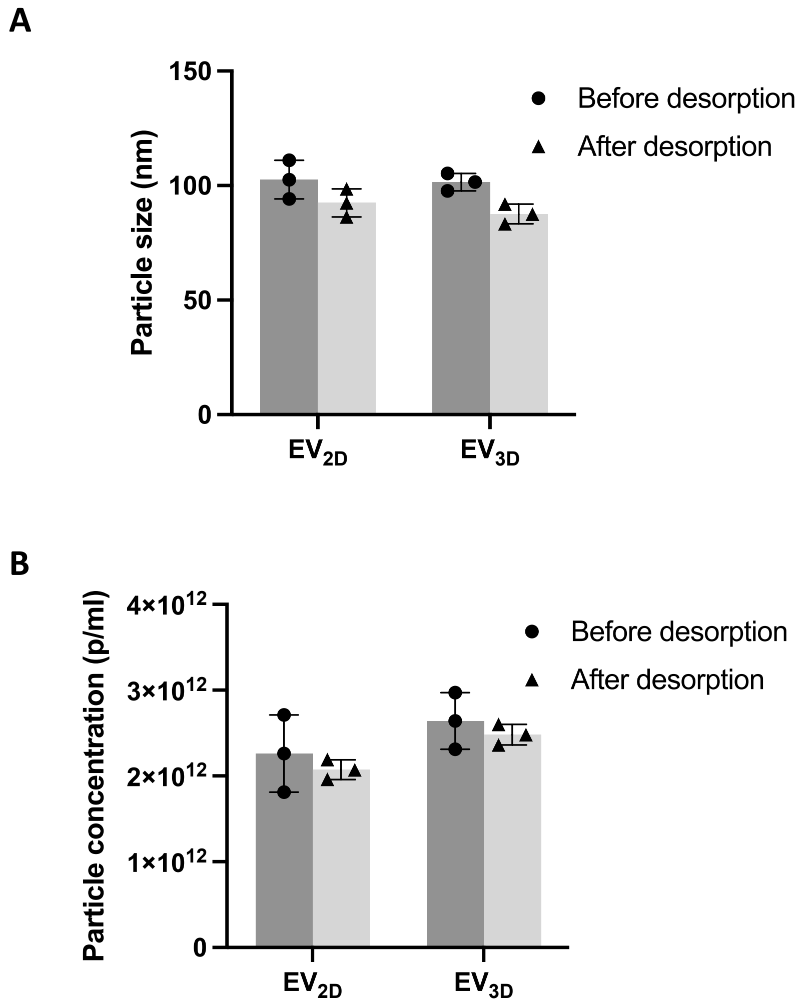

Figure S4: Protein corona desorption of non-incubated EVs. Changes caused by desorption **(A)** particle size (n=3, biologically independent samples) **(B)** particle concentration (n=3, biologically independent samples). Non-FBS incubated EVs subjected to desorption protocol experienced no significant changes in particle number when subjected to desorption protocol suggesting no disintegration or aggregation of EVs; thus, the suitability of the protocol to be applied in the HC analysis study. Data were obtained by two-tailed paired t-test analysis with Holm-Šídák test ($p = 0.05$) and are presented as mean $\pm$ SD.

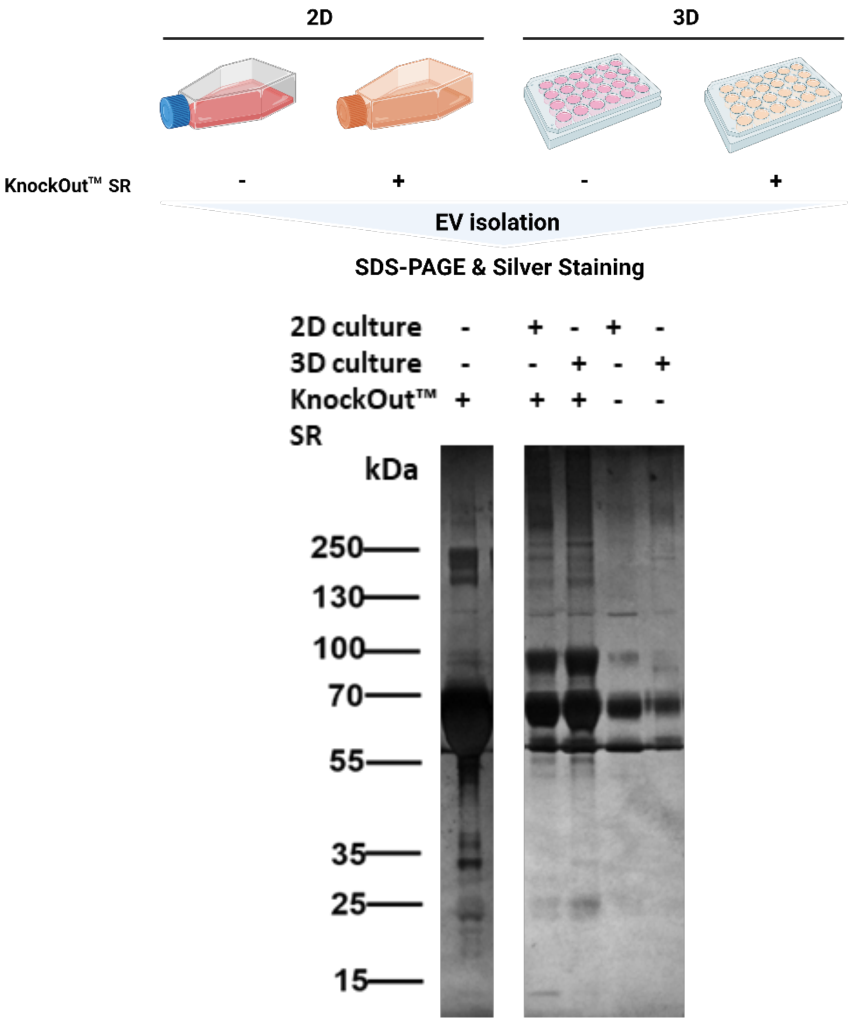

Figure S5: The effect of cell architecture on the protein identity of EVs. MSCs were cultured for 24 h in 2D and 3D conditions in the basal media w/ and w/o KnockOut™ SR, followed by CCM harvest for EV isolation. Silver-stained SDS-PAGE of EVs isolated from all conditions was performed (n=1). The results confirmed that similar proteins could be qualitatively acquired from the media during the culturing independent of the cell's architecture.

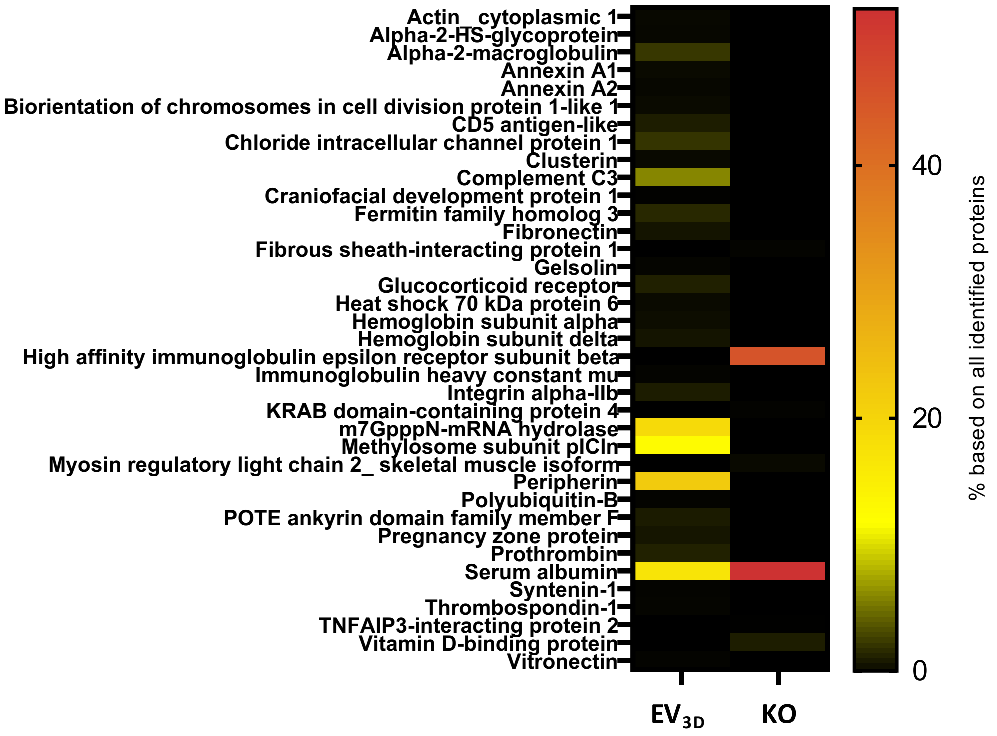

Figure S6: Heatmap of the most abundant proteins detected in non-incubated EV_{3D} comparing with Knockout™ serum replacement (KO). m7GpppN-mRNA hydrolase, methylosome subunit plCln, peripherin, and serum albumin, were the most abundant proteins found in EV_{3D}. However, only serum albumin was acquired from KO.

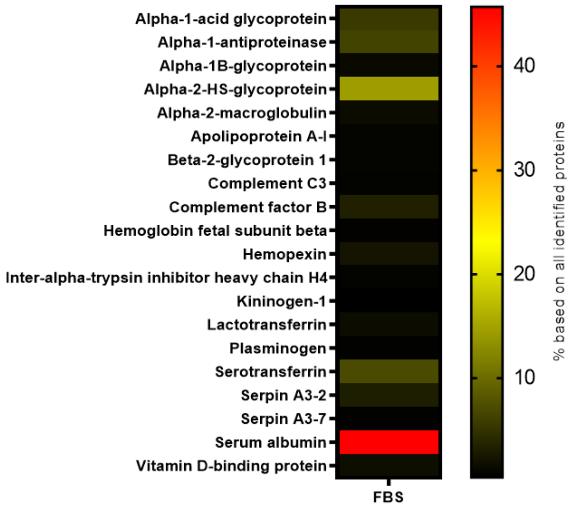

Figure S7: Heatmap of the most abundant proteins detected in EV-D FBS by LC-MS. EV-D FBS was analysed against a bovine database. In total 31 proteins were identified. Top 20 proteins identified are displayed in the heatmap. Serum albumin was the most abundant protein and was found to adsorb on the EV surface as protein corona upon FBS incubation.

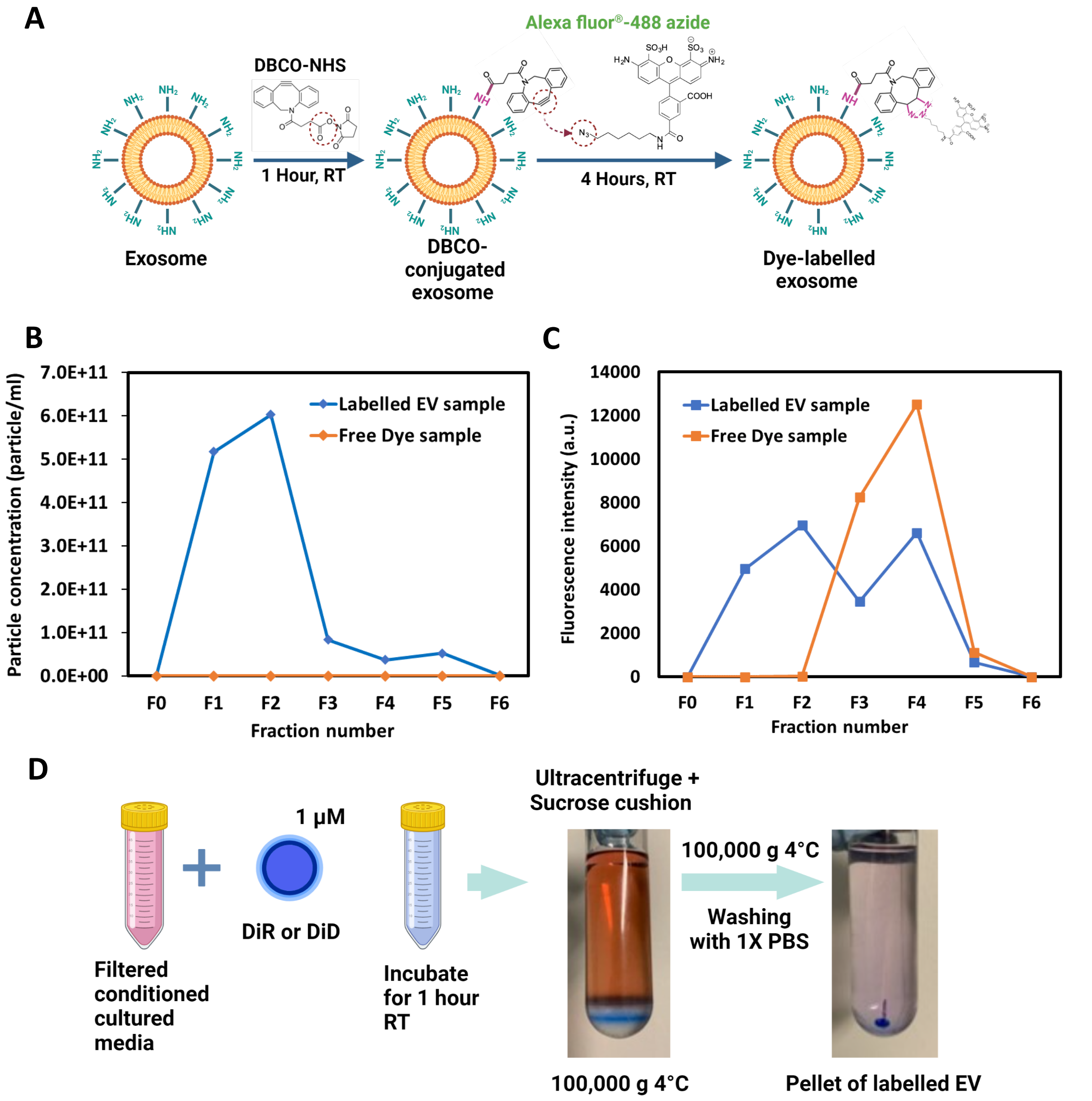

Figure S8: EV labelling for *in vitro* cellular uptake, *in vivo* biodistribution, and *in vivo* cellular uptake. **(A)** EV labelling using copper-free click chemistry for *in vitro* cellular uptake. **(B, C)** Purification of EV labelled by copper-free click chemistry using gel filtration on a sepharose CL-2B column by particle number **(B)** and fluorescence intensity **(C)**. Labelled EVs can be obtained in fraction 1 and 2. **(D)** EV labelling using lipophilic dye incorporation and purification for *in vivo* biodistribution and *in vivo* cellular uptake. *The figure was created with BioRender.com.*

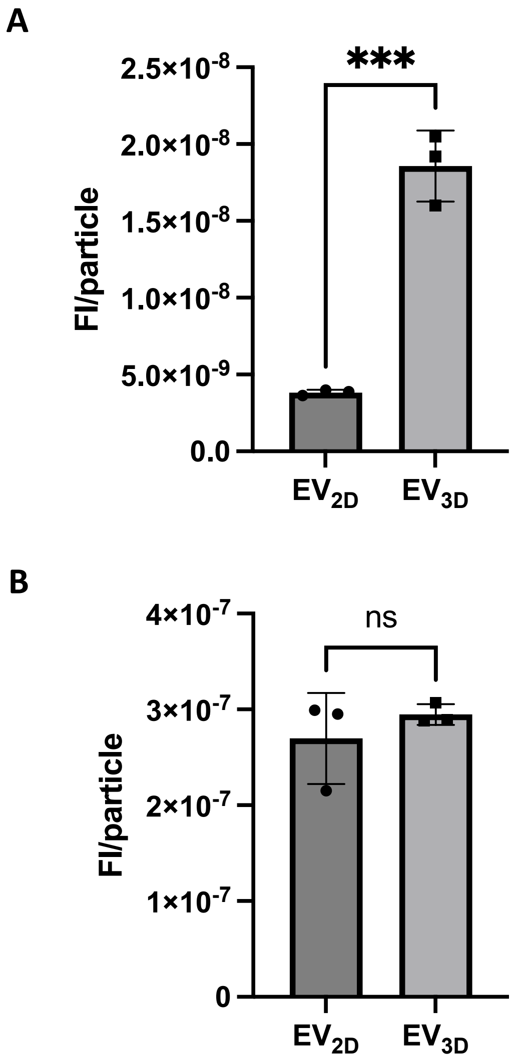

Figure S9: Labelling efficiency of EVs calculated as FI/particle. EVs labelled by **(A)** AF488 azide using DBCO-NHS linker and copper-free click chemistry (***p=0.0004), and **(B)** Lipophilic dye incorporation (1 µm DiD), were subjected to NTA and fluorescence intensity (FI) measurement by plate reader to derive FI per particle. Data are presented as mean ± SD (n = 3, biologically independent samples) with two-tailed unpair t-Test statistical analysis (ns: no significance).

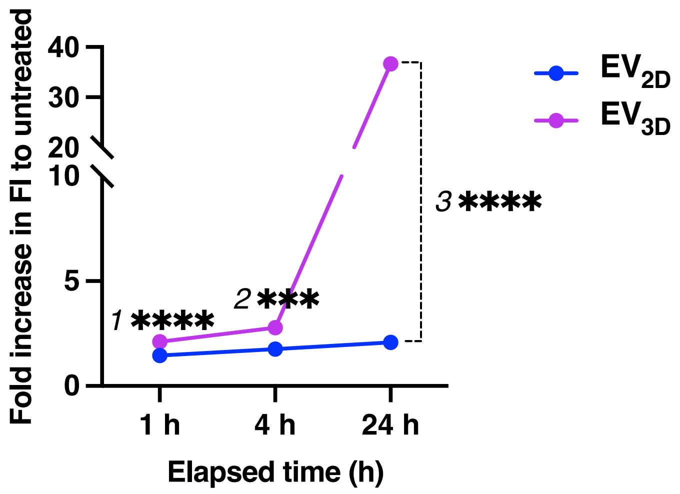

Figure S10: Cellular uptake of DiD-labelled EVs HepG2 cells. DiD labelled EVs were incubated with cells, with the presence of 10% FBS, at the dose of 2 x 10^{10} particles per well (24-well plate) for 1h, 4h, and 24h. Cellular uptake was measured by flow cytometry, and uptake was expressed as a fold increase of the mean DiD signal per cell (MFI) compared to untreated cells. Time course and dose dependence on the uptake were shown. EV_{3D} was more preferential for HepG2 uptake. Data were obtained by two-tailed unpaired t-Test analyses with the Holm-Šídák test and are presented as mean ± SD (n = 3, [1]****p=7x10^{-6}, [2]***p=3x10^{-4}, and [3]****p=2x10^{-6}).

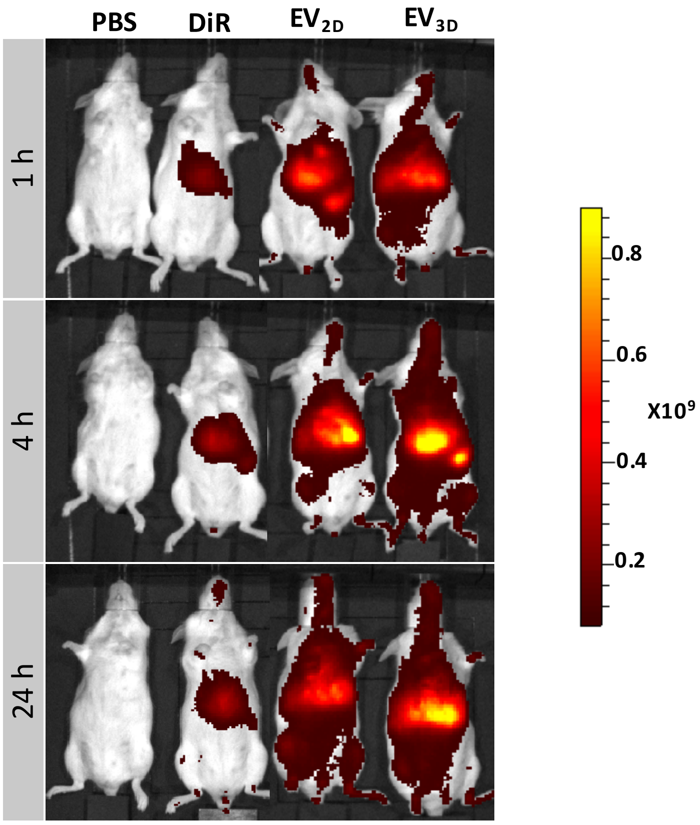

Figure S11: Whole-body live (ventral) imaging showing EV biodistribution at 1, 4, and 24 hours post IV injection. Animals were intravenously injected with 2×10^{11} DiR-labelled EVs, PBS, or free dye (control). EV_{2D} shows shorter half-life when compared to EV_{3D}.

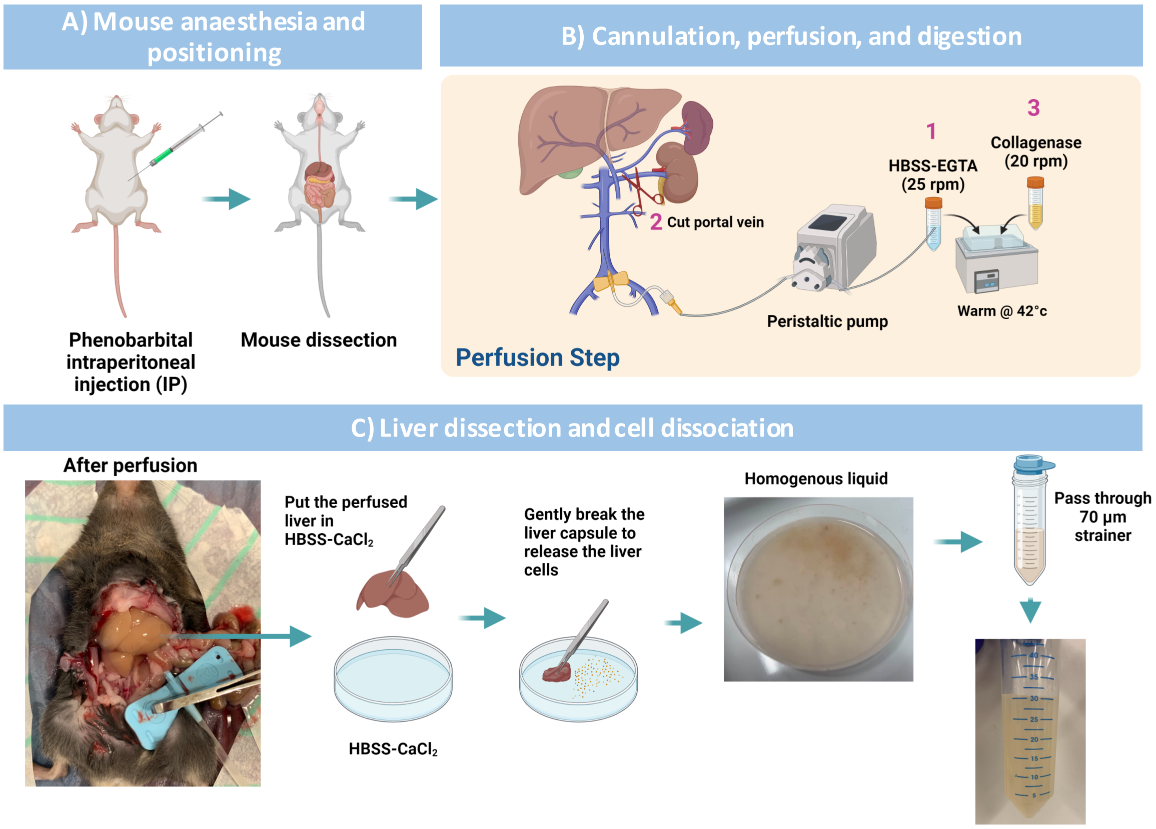

Figure S12: Step-by-step illustration of isolation of liver perfusion and liver isolation. (A) Mouse anaesthesia (with phenobarbital IP) and mouse positioning. **(B)** Cannulation into inferior vena cava followed by perfusion with HBSS resulting in a pale, enlarged liver. Digestion was then performed using collagenase type IV solution. **(C)** Liver dissection and cell dissociation: Cell suspension was taken for further fractionation using differential centrifugation. *The figure was created with BioRender.com.*

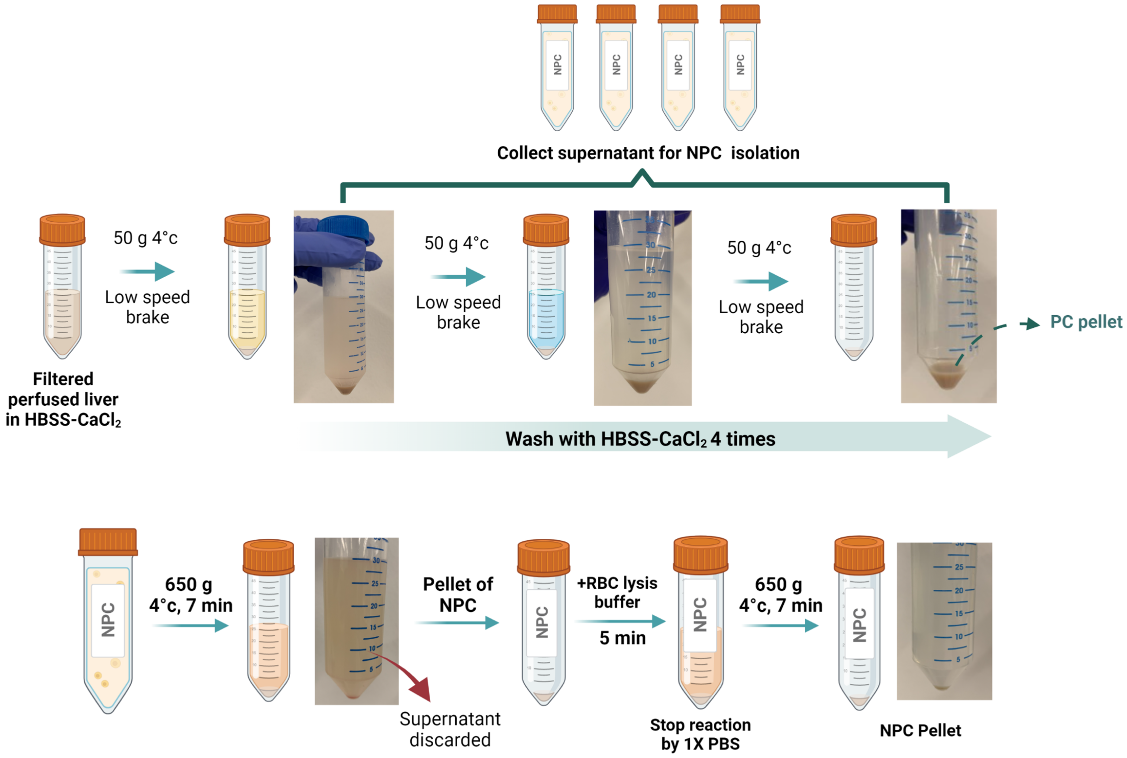

Figure S13: Schematic presentation of the differential centrifugation protocol used for liver cell subtype separation and enrichment for subsequent analysis. Parenchymal liver cells (PC) majorly are hepatocytes, while non-parenchymal cells comprise Kupffer cells, endothelial cells, and stellate cells. Cell sub-populations were further stained with specific antibodies and characterised by flow cytometry. *The figure was created with BioRender.com.*

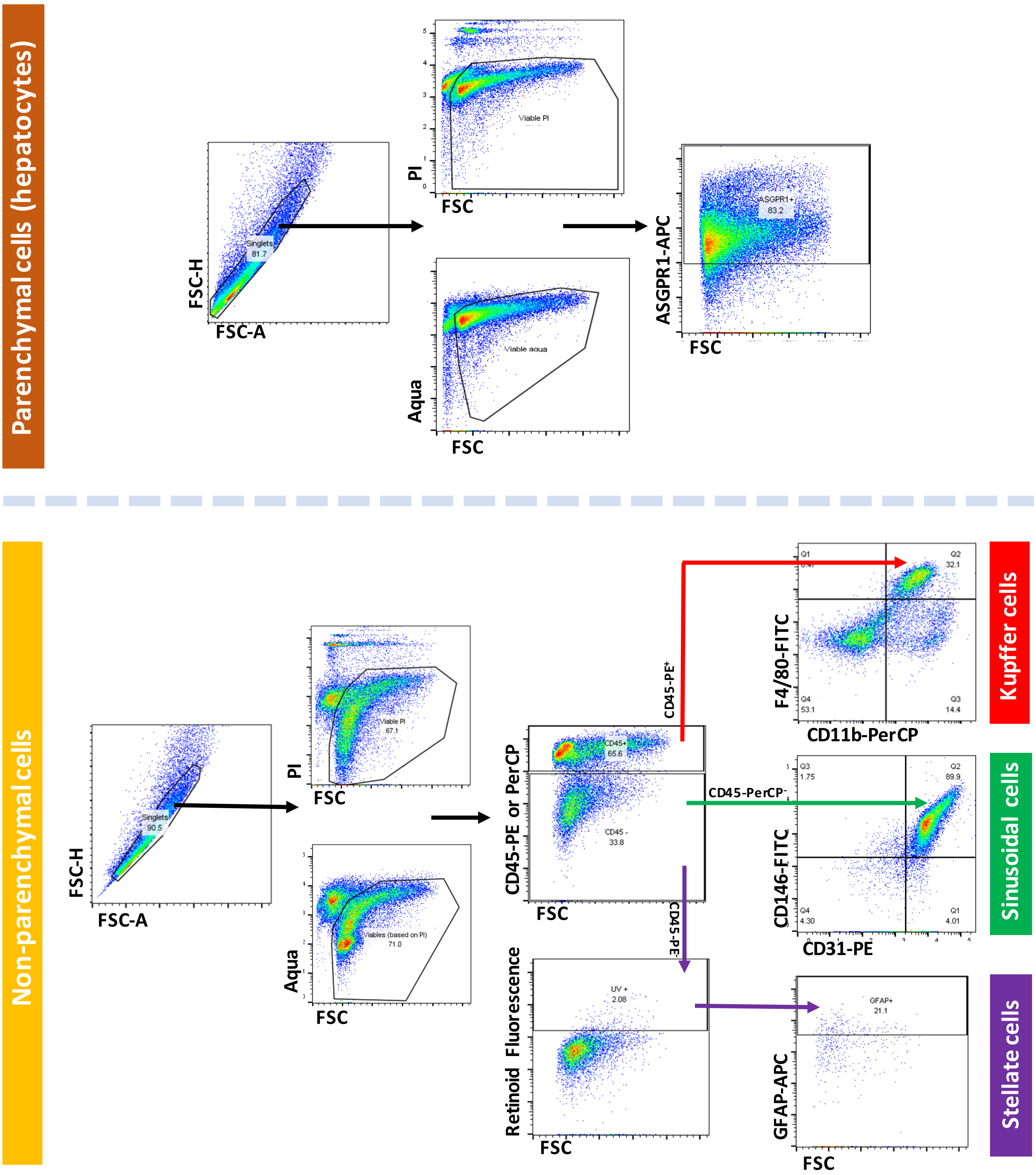

Figure S14: Identification of parenchymal cells and non-parenchymal cells by immune staining and flow cytometry. Liver nonparenchymal cells from C57BL/6 mouse were isolated and enriched by differential centrifugation. Cells in the different fractions were stained with viability dye (Zombie Aqua, PI was also used to validate the gating strategy for viable cells.), CD45, F4/80, CD11b, CD146, CD31, and GFAP. Parenchymal cells (hepatocytes) were identified as ASGPR1^{+}. Kupffer cells were identified as CD45^{+}, F4/80^{+}, and CD11b^{+}. Liver sinusoidal cells were identified as CD45^{-}, CD146^{+}, and CD31^{+}. Hepatic stellate cells were identified as CD45$^{-,}$ retinoid autofluorescence (sorted by violet laser 405 nm), and GFAP^{+}.

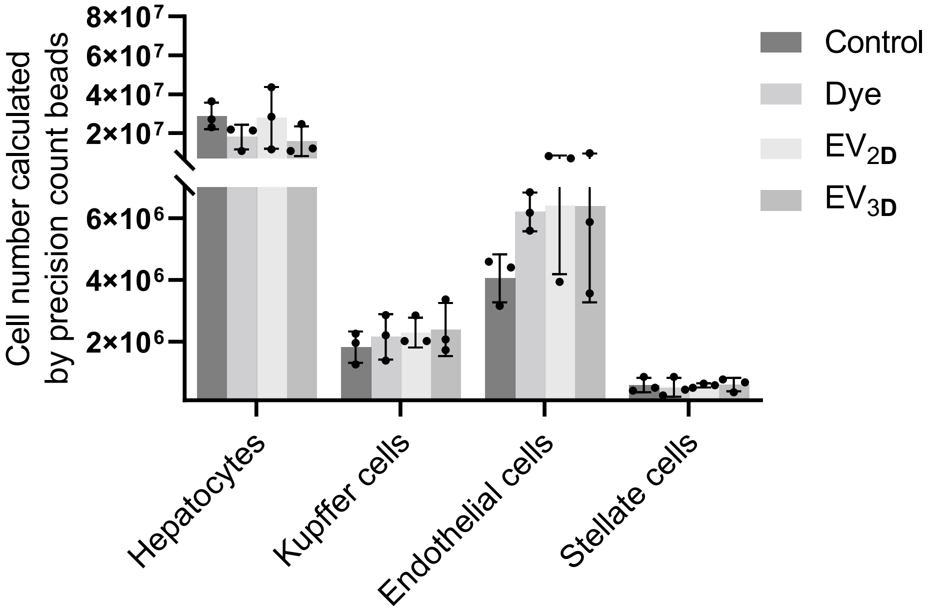

Figure S15: Cell number of each liver sub-population isolated from the different mice to confirm protocol reproducibility (n=3, biologically independent animals)**.** Data are presented as mean ± SD.

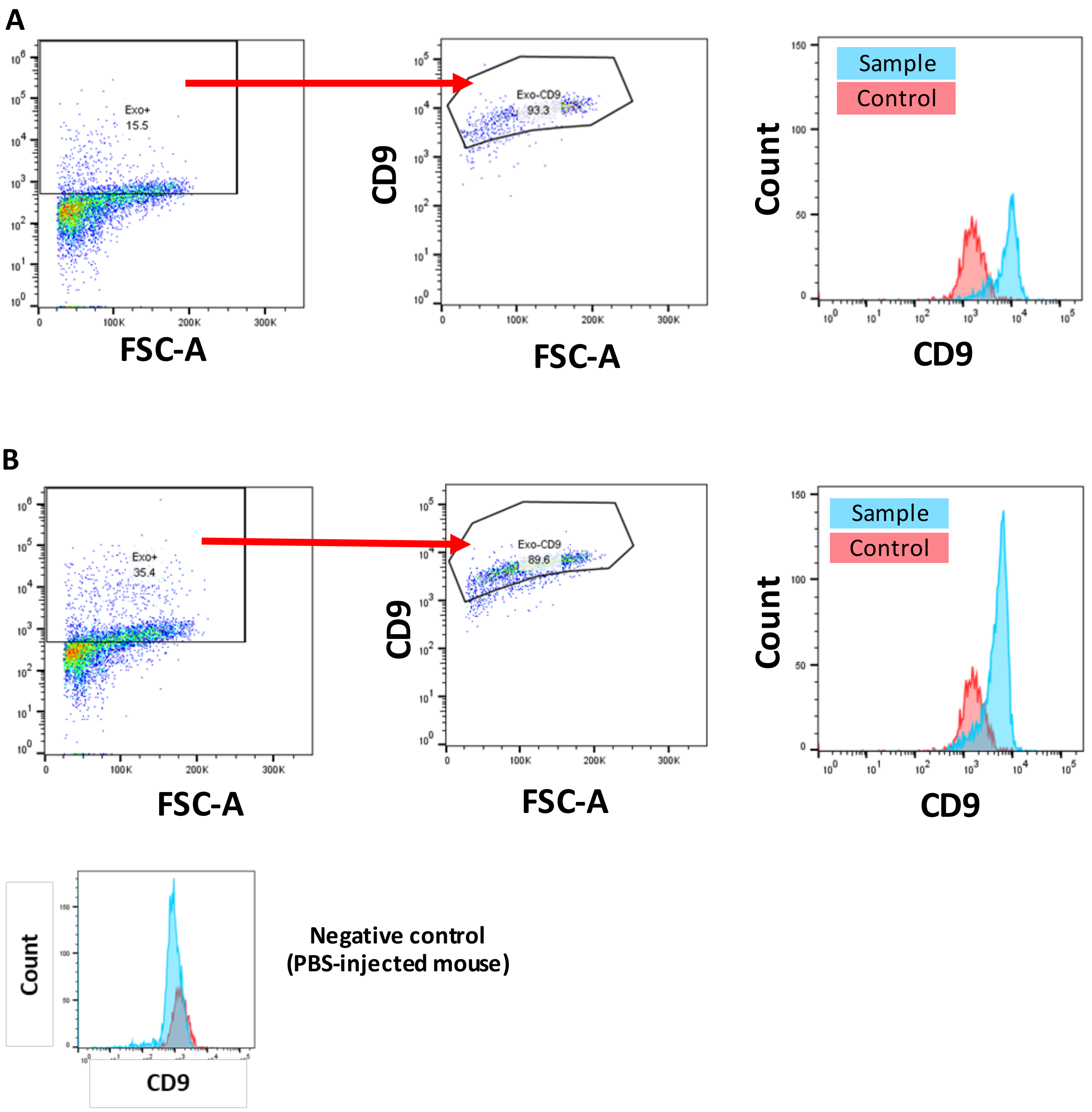

Figure S16: Confirmation of *in vivo* uptake of MSC EVs by hepatocyte by staining of EV surface marker. The parenchymal cells isolated from a mouse injected with **(A)** EV_{2D} **(B)** EV_{3D} were subjected to intracellular staining with anti-human CD9 to confirm that uptake of EV was not a DiD labelling artefact.

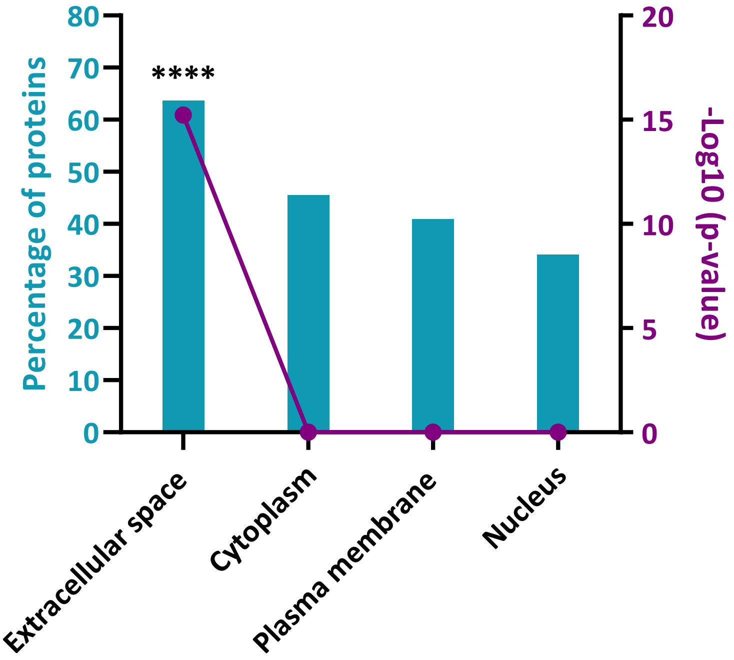

Figure S17: Gene Ontology (GO) analysis on the cellular component aspect for classification of the protein identified on EV by LC-MS. GO analysis were performed using FunRich® software v.3.1.3 to classify EV proteins based on the cellular component. Extracellular space proteins (enriched with ****$p=5.89x10^{-16}$) were further analysed for a biological process aspect as shown in **Figure 5A**.

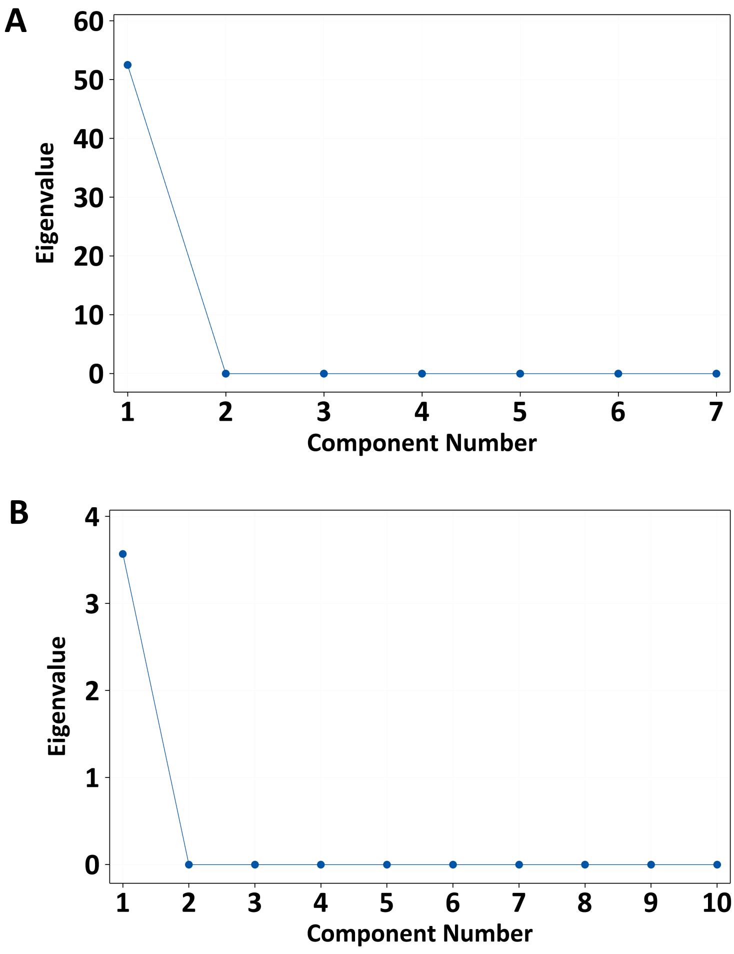

Figure S18: Scree plots of the principal component analysis. To check whether PCA is applicable for the data, the scree plot is established to display how much variation each PC capture from the data. PC1 is potentially extracted all the variances **(A)** for PCA of EV shown in **Figure 5C**. **(B)** for PCA of HC shown in **Figure 5D**.

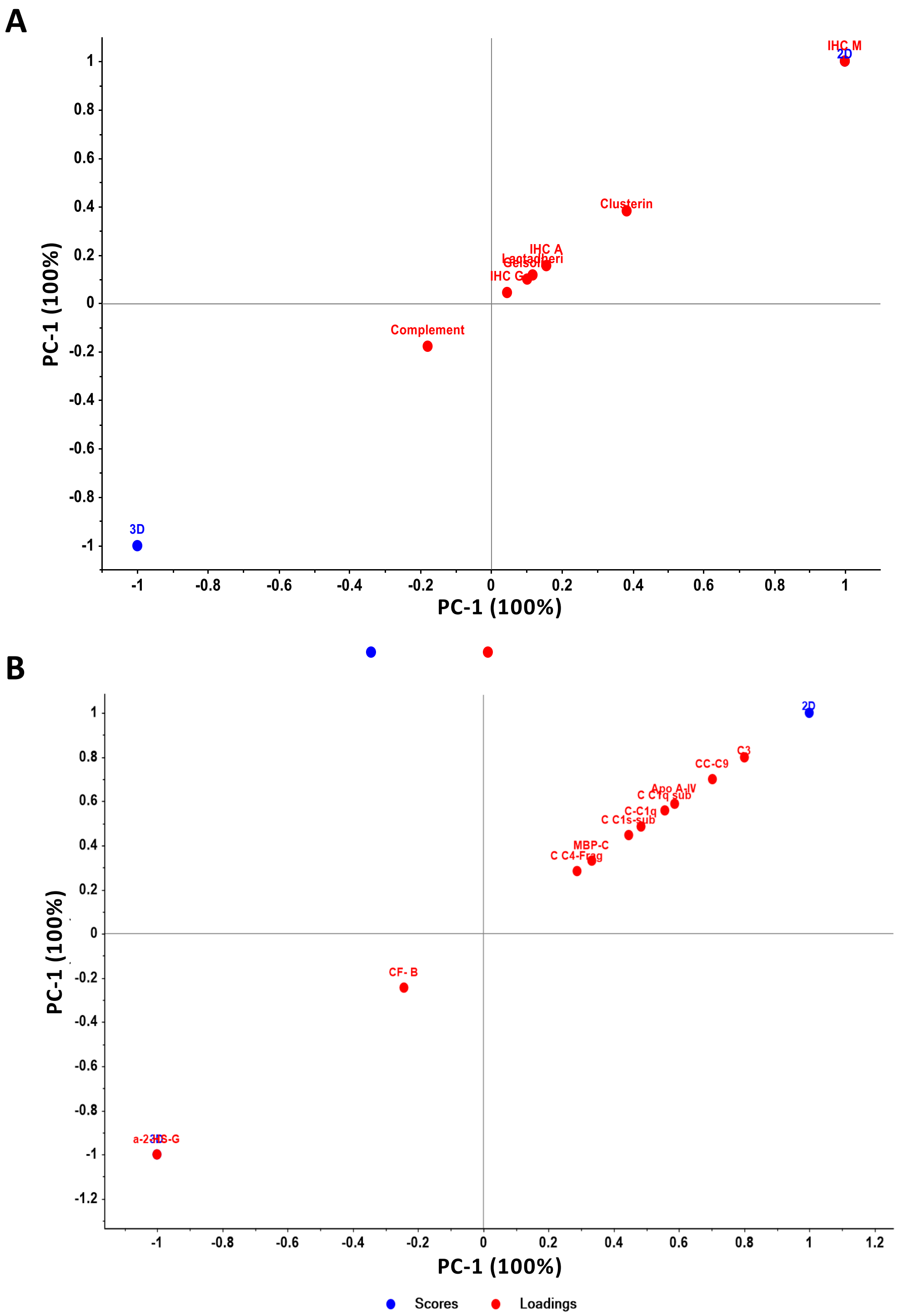

Figure S19: PCA Bi-plots showing both PC scores and loadings of variables. As PC1 is potentially extracted all the variances (100%), data were plotted against PC1 vertically and horizontally to interpret relationships between protein corona and **(A)** EV_{2D} & EV_{3D}, **(B)** HC_{2D} & HC_{3D}.

Abbreviations: IHC A; Immunoglobulin heavy constant a, IHC G; Immunoglobulin heavy constant g, IHC M; Immunoglobulin heavy constant m, C3; Complement C3, CF- B; Complement factor B, C C1s-sub; Complement C1s subcomponent, a-2-HS-G; Alpha-2-HS-glycoprotein, CC-C9; Complement component C9, Apo A-IV; Apolipoprotein A-IV, C-C1q; Complement C1q subcomponent, MBP-C; Mannose-binding protein C, C C4-Frag; Complement C4 (Fragments), C C1q sub s_1; Complement C1q subcomponent s_1

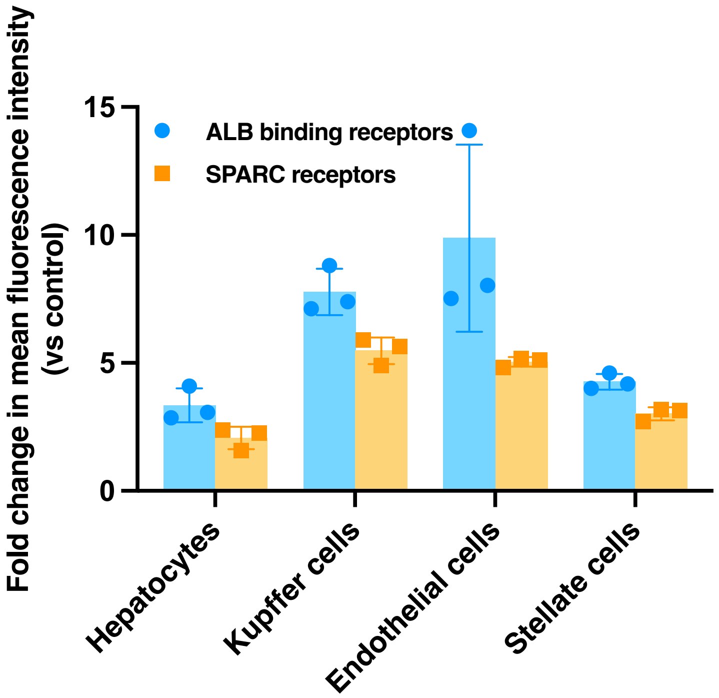

Figure S20: Abundance of albumin-binding and SPARC receptors in liver cell sub-populations. Fractions of liver cell sub-populations (n=3, biologically independent animals) were stained with antibodies for cell characterisation, BSA labelled with Cy5 (0.25 µg per 10^6 cells in 100 µl volume), and anti-SPARC (a subset of albumin-binding receptors), prior to analysis by flow cytometry. Data are presented as mean ± SD.

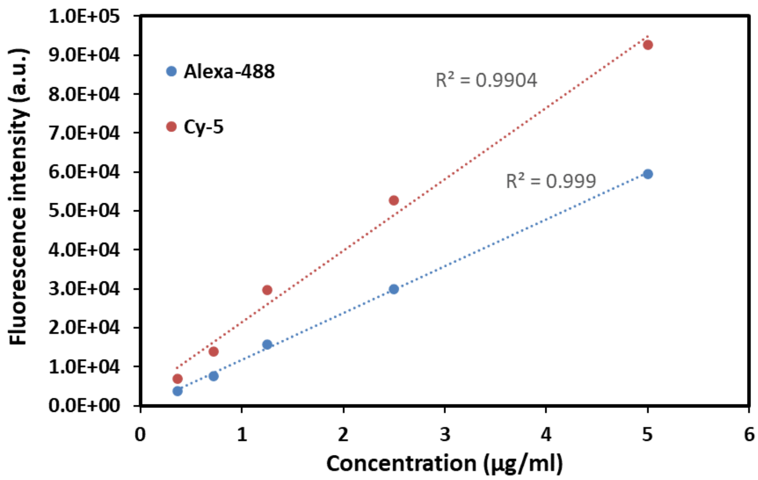

Figure S21: Standard curve of Alexa-fluor 488 spiked with the same range of the concentrations of Cy5. The standard curves were prepared to validate the measurement of fluorescence intensity of the samples co-stained with two fluorophores.

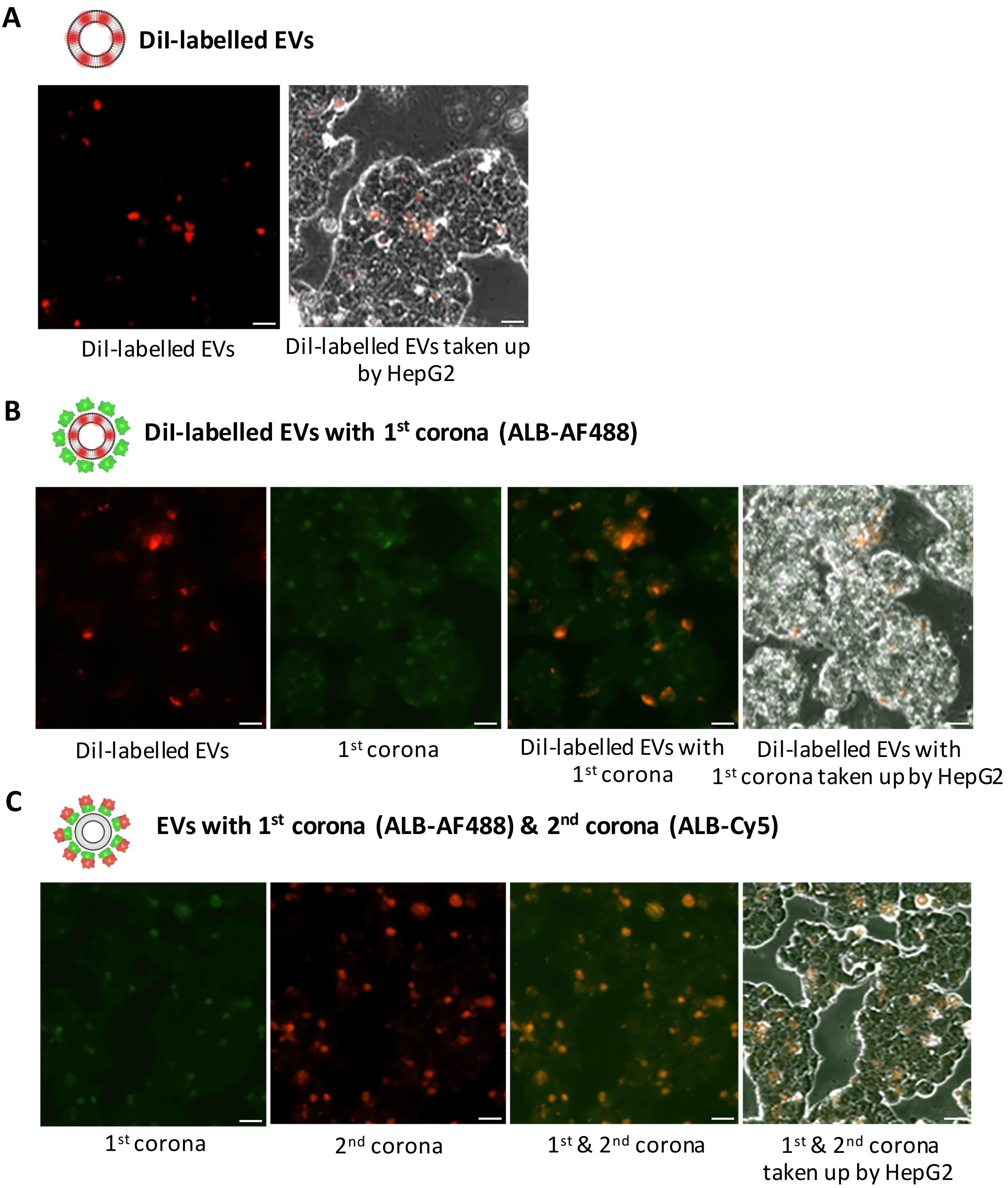

Figure S22: Fluorescence microscopy images of HepG2 cells treated with EV w/ and w/o the albumin-enriched protein corona for 24 h. HepG2 cells treated with **(A)** DiI-labelled EV, **(B)** DiI-labelled EV enriched with AF488-albumin primary protein corona, **(C)** EV enriched with AF488-albumin primary corona and Cy5-albumin secondary corona. Cells were imaged 24 h after incubation in a Nikon epifluorescence microscope TS2R. Co-localisation of EVs and primary corona or primary and second corona is shown by the yellow colour. (n=1, scale bar 20 µm)

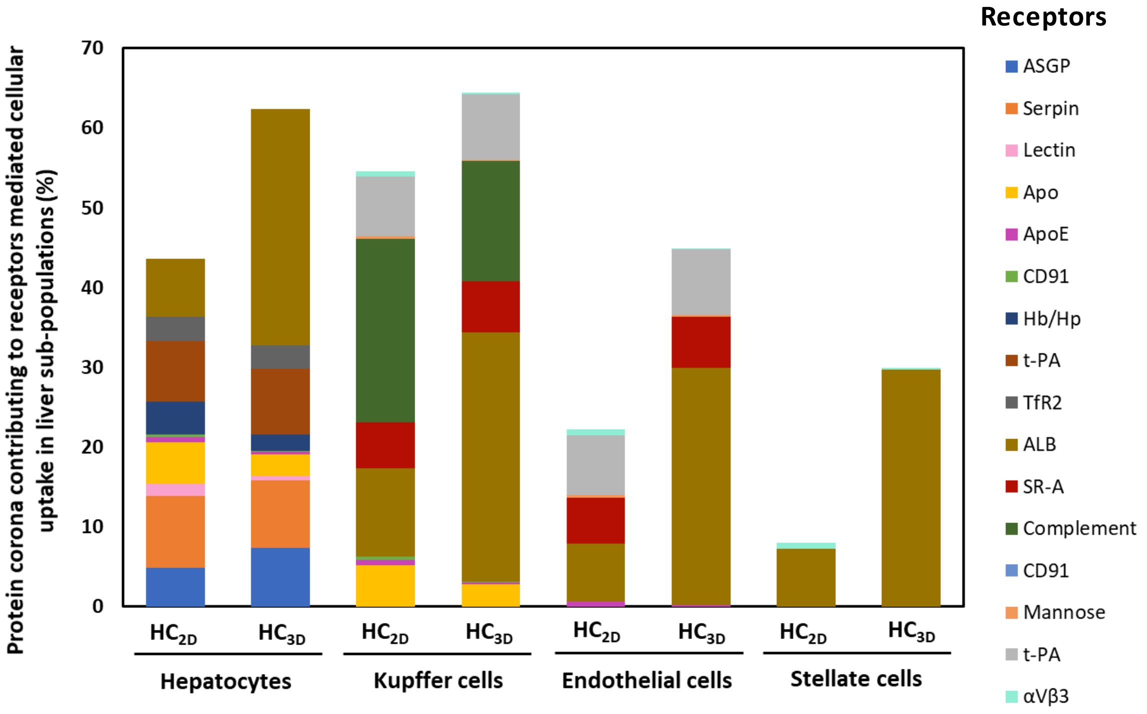

Figure S23: The contribution of hard protein corona on the EV uptake by liver sub-population. To map HC proteins with known receptors expressed on the different types of liver cells, distribution of typical plasma protein receptors on each liver sub-population and their protein ligands was thoroughly reviewed (*see* Table S9). The contribution to the cellular uptake by each cell type was evaluated by quantitatively recapitulating ligand-receptor matching as shown. Albumin-binding receptors (ALB RC) express in all sub-populations and possibly most contributed to the preferential uptake of EV_{3D}.

[1]ALB is referred to various types of receptors those can bind albumin, i.e., gp60, gp30, gp18, Fc Rn, TGF-β, SPARC.

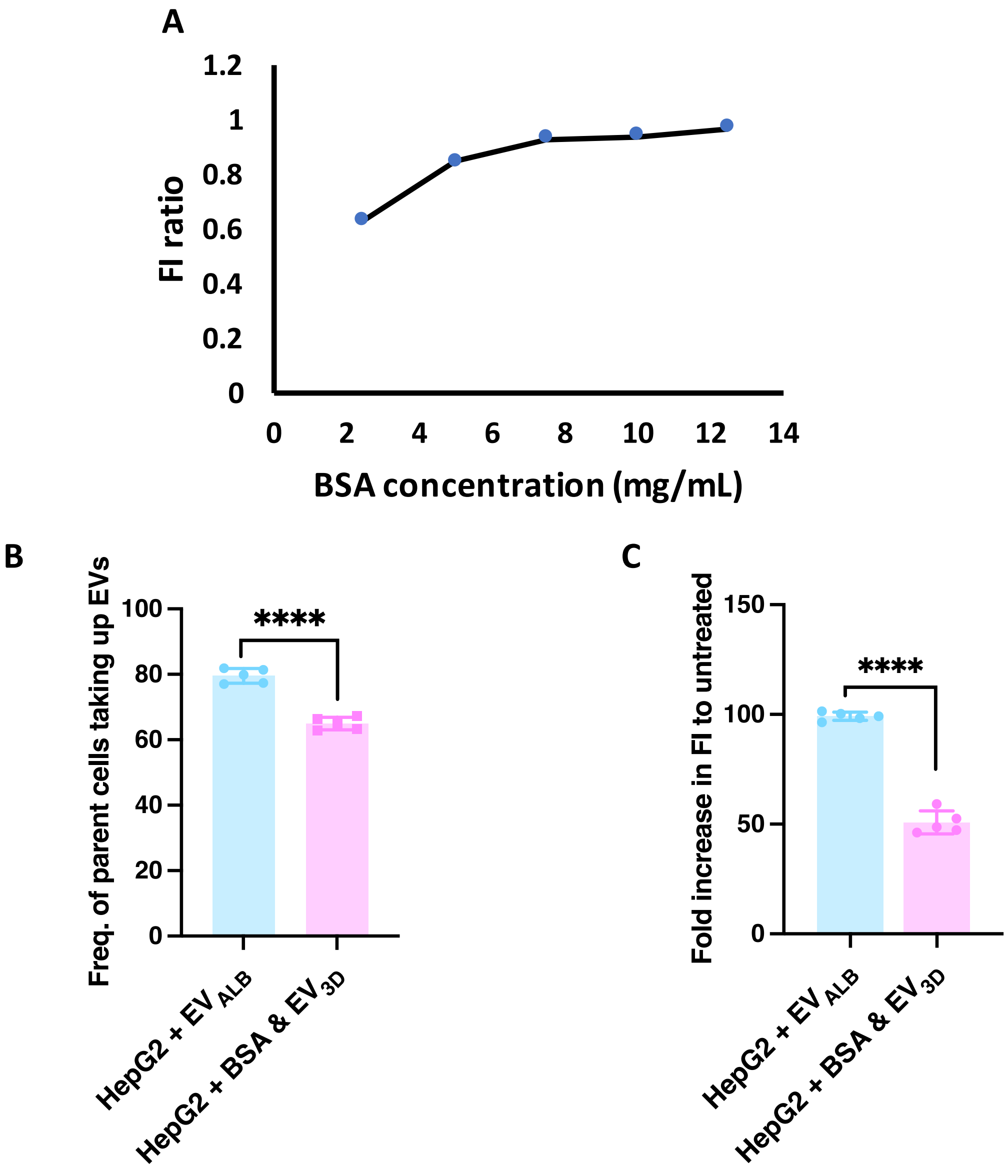

Figure S24: Albumin receptors blocking study *in vitro*. (A) Uptake of fluorescently labelled BSA in HepG2 to determine BSA concentration to be used in blocking studies. Uptake of EV_{3D} in the presence and absence of excess BSA as frequency of EVs **(B)** positive cells (****$p<1x10^{-6}$) and **(C)** fold increase in MFI to untreated (****$p=4x10^{-6}$). Data are presented as mean ± SD (n = 5, biologically independent samples) with two-tailed unpaired t-Test statistical analysis (p**** < 0.0001).

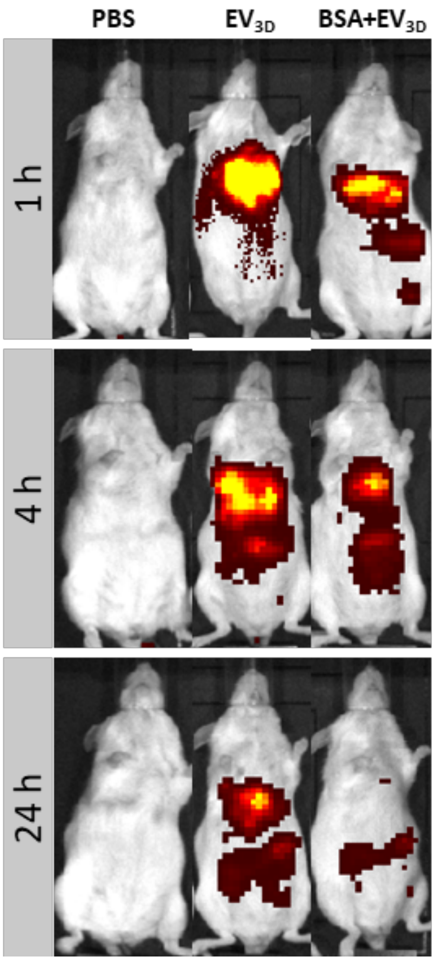

Figure S25: Whole-body live (ventral) imaging showing the effect of BSA pre-administration on EV_{3D} biodistribution at 1, 4, and 24 hours post IV injection. *In vivo* uptake of EV_{3D} in the liver is reduced when mice were pre-injected with BSA (5 min pre-EV injection, 10 mg/ml, 100 µl) prior IV injection of DiR-labelled EV_{3D} (2×10^{11} particles/mouse). It is possible that the circulation times of EVs are prolonged though additional quantitative studies are required.

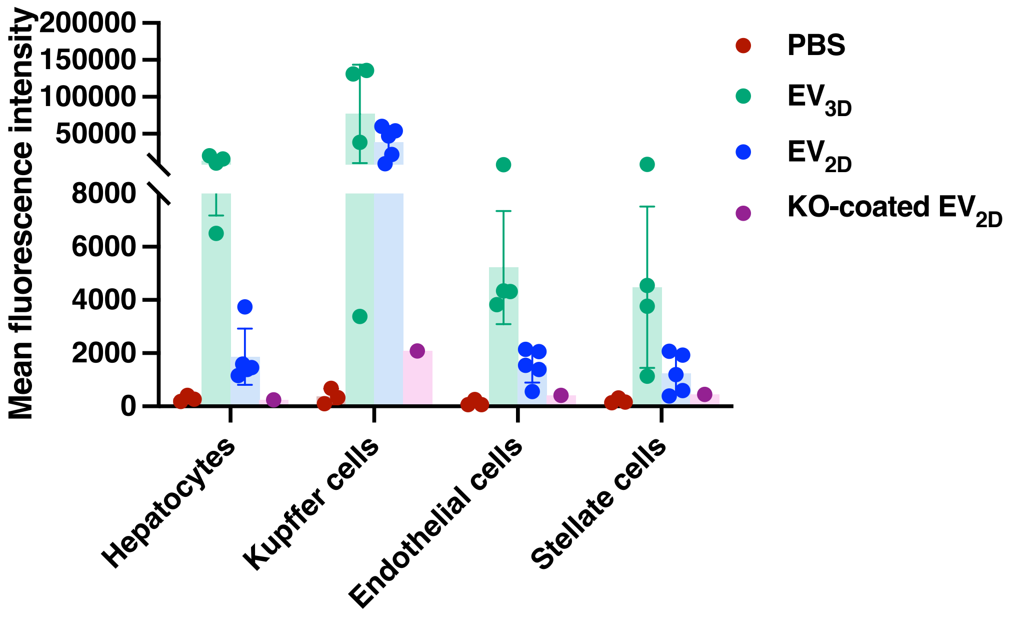

Figure S26: *In vivo* uptake of EVs by liver sub-population confirming the non-contributory effects of KO in EV uptake improvement. EV_{2D} were incubated with KO for 24 h and subjected to washing by ultracentrifugation to eliminate unbound KO proteins, followed by intravenous injection (n=1). This further confirms that the enhanced EV internalization can be mediated mainly by albumin, not the other KO media ingredients. Data are presented as mean ± SD.

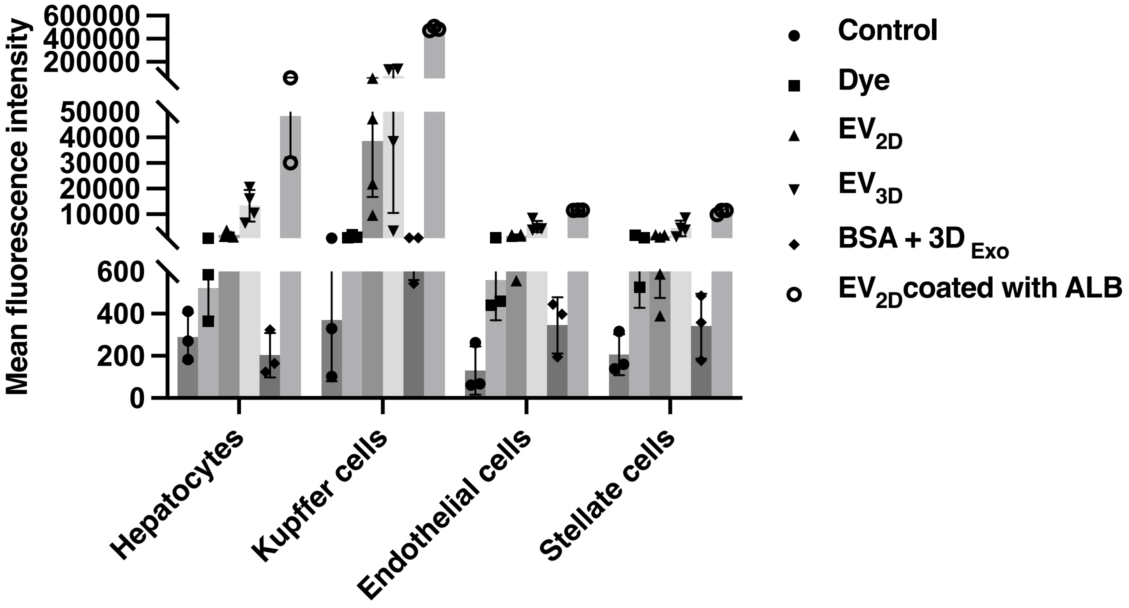

Figure S27: Summary of *In vivo* uptake of various types of EVs by liver sub-population (n ≥ 3, biologically independent animals, data are presented as mean ± SD.)

Supplementary Tables

Table S1. Protein corona desorption efficiency for liposomes and EVs.

Samples	FBS incubation (+/-)	Protein bound/surface area (mg/m^2)[1,3]	% Desorption efficiency[2,3]
Liposomes	+	3.25 ± 0.02	94.52 ± 0.45
Liposomes-PEG	+	0.38 ± 0.05	95.21 ± 1.56
PANC-1 EV	-	3.05 ± 0.53	n/a
	+	5.53 ± 0.72	95.05 ± 0.85
MSC EV_{2D}	-	0.62 ± 0.07	n/a
	+	1.7 ± 0.29	95.54 ± 4.76
MSC EV_{3D}	-	1.02 ± 0.042	n/a
	+	1.56 ± 0.17	94.16 ± 2.65

[1] Calculated using formula A/0.05 (for non-FBS incubated samples) or B/0.05 (for FBS incubated samples); A: protein amount in EV sample, B: protein amount in HC-EV sample
[2] % Desorption was calculated as (C/B-A) x 100; A: protein amount of EV, B: protein amount of HC-EV, C: protein amount in the desorbed protein corona
[3]Values are expressed as mean ± SD, where n=3

Table S2. Loss of intrinsic EV proteins during the desorption step.

Samples[1]	Loss percentage (%) [2,3]
PANC-1 EV	4.75 ± 0.90
MSC EV_{2D}	4.36 ± 0.51
MSC EV_{3D}	4.78 ± 0.18

[1]Non-incubated EVs were subjected to desorption in order to evaluate the ability to desorb intrinsic proteins, considered as a protocol disadvantage.
[2] % Loss of EV intrinsic protein due to desorption process was calculated as (C/A) x 100; A: protein amount in EV sample, C: protein amount in the desorbed protein corona sample.
[3]Values are expressed as mean ± SD (n=3).

Table S3. Quantitative analysis of proteins in non-incubated MSC EV samples.

Gene code	Description	Average in %[1,2,3]	
		EV_{3D}	EV_{2D}
ACTA1	Actin_ alpha skeletal muscle	0.019 ± 0.001	0.600 ± 0.389
ACTB	Actin_ cytoplasmic 1	0.329 ± 0.016	9.977 ± 1.690
AHSG	Alpha-2-HS-glycoprotein	0.289 ± 0.013	1.110 ± 0.025
A2M	Alpha-2-macroglobulin	2.551 ± 0.028	4.467 ± 1.455
ANXA1	Annexin A1	0.433 ± 0.044	0.317 ± 0.092
ANXA2	Annexin A2	0.299 ± 0.008	3.036 ± 2.126
ANXA5	Annexin A5	0.193 ± 0.001	1.177 ± 0.098
BOD1L1	Biorientation of chromosomes in cell division protein 1-like 1	0.444 ± 0.006	0.341 ± 0.141
CD5L	CD5 antigen-like	1.364 ± 0.022	0.290 ± 0.208
CLIC1	Chloride intracellular channel protein 1	2.447 ± 0.049	3.328 ± 2.094
CLU	Clusterin	0.390 ± 0.005	3.931 ± 0.163
C3	Complement C3	6.299 ± 0.155	4.648 ± 2.452
CFDP1	Craniofacial development protein 1	0.051 ± 0.005	1.875 ± 1.090
FERMT3	Fermitin family homolog 3	1.841 ± 0.163	1.735 ± 1.057
FN1	Fibronectin	0.936 ± 0.022	3.404 ± 1.903
LGALS3BP	Galectin-3-binding protein	0.050 ± 0.003	1.707 ± 0.420
GSN	Gelsolin	0.218 ± 0.008	1.163 ± 0.169
NR3C1	Glucocorticoid receptor	1.473 ± 0.013	2.657 ± 1.011
GAPDH	Glyceraldehyde-3-phosphate dehydrogenase	0.041 ± 0.004	1.479 ± 1.025
HP	Haptoglobin OS=Homo sapiens	0.035 ± 0.001	1.000 ± 0.789
HSPA6	Heat shock 70 kDa protein 6	0.451 ± 0.002	1.646 ± 0.960
HSPA8	Heat shock cognate 71 kDa protein	0.082 ± 0.001	0.812 ± 0.180
HSP90AA2P	Heat shock protein HSP 90-alpha A2	0.063 ± 0.003	0.500 ± 0.364
HBA1	Hemoglobin subunit alpha	0.567 ± 0.023	2.970 ± 0.490
HBD	Hemoglobin subunit delta	0.916 ± 0.002	4.204 ± 0.776
IGHA1	Immunoglobulin heavy constant alpha 1	0.268 ± 0.004	1.709 ± 0.261
IGHG3	Immunoglobulin heavy constant gamma 3	0.017 ± 0.001	0.441 ± 0.588
IGHM	Immunoglobulin heavy constant mu	0.147 ± 0.004	9.388 ± 1.404
INTS13	Integrator complex subunit 13	0.205 ± 0.004	0.755 ± 0.368
ITGA2B	Integrin alpha-IIb	1.278 ± 0.011	3.333 ± 1.618
MFGE8	Lactadherin	0.171 ± 0.004	1.258 ± 0.393
DCP2	m7GpppN-mRNA hydrolase	19.258 ± 0.495	0.305 ± 0.086
CLNS1A	Methylosome subunit pICln	12.779 ± 0.419	2.936 ± 2.423
PRPH	Peripherin	22.111 ± 0.124	1.621 ± 0.295
UBB	Polyubiquitin-B	0.154 ± 0.003	2.669 ± 1.008
POTEF	POTE ankyrin domain family member F	1.259 ± 0.012	1.433 ± 0.138
PZP	Pregnancy zone protein	0.976 ± 0.013	2.146 ± 0.390
FAM102A	Protein FAM102A	0.107 ± 0.002	0.673 ± 0.169
F2	Prothrombin	1.523 ± 0.037	2.936 ± 0.800
PCP4L1	Purkinje cell protein 4-like protein 1	0.035 ± 0.001	0.079 ± 0.057
PKM	Pyruvate kinase PKM	0.094 ± 0.002	1.474 ± 0.375
TF	Serotransferrin	0.002 ± 0.000	0.309 ± 0.276
ALB	Serum albumin	17.293 ± 0.234	3.876 ± 1.500
SDCBP	Syntenin-1	0.164 ± 0.006	0.422 ± 0.091
THBS1	Thrombospondin-1	0.227 ± 0.008	2.167 ± 0.340
VTN	Vitronectin	0.150 ± 0.006	1.697 ± 0.860

[1]The percentage was calculated based on all identified proteins (46 proteins) by LC-MS
[2]Data were analysed using Homo sapiens (human) database.
[3]Values are expressed as mean ± SD (n=3).

Table S4. Quantitative analysis of proteins of FBS-incubated MSC EV samples.

Gene code	Description	Average in %[1,2,3] EV_{3D-1}	EV_{3D-2}	EV_{2D-1}	EV_{2D-2}
A2M	Alpha-2-macroglobulin	1.189 ± 0.024	1.000 ± 0.006	1.956 ± 0.033	2.038 ± 0.058
C3	Complement C3	1.114 ± 0.014	1.117 ± 0.005	2.224 ± 0.008	2.300 ± 0.015
ALB	Serum albumin	27.328 ± 0.223	22.407 ± 0.111	4.398 ± 0.064	4.154 ± 0.049
TF	Serotransferrin	2.570 ± 0.012	2.625 ± 0.027	2.713 ± 0.008	2.450 ± 0.005
ITIH4	Inter-alpha-trypsin inhibitor heavy chain H4	2.504 ± 0.009	2.774 ± 0.022	4.344 ± 0.013	4.417 ± 0.034
F2	Prothrombin	0.921 ± 0.007	0.863 ± 0.006	1.916 ± 0.024	1.900 ± 0.016
CFB	Complement factor B	6.984 ± 0.052	8.691 ± 0.082	7.914 ± 0.068	7.061 ± 0.135
SERPINA1	Alpha-1-antiproteinase	2.895 ± 0.022	3.395 ± 0.022	2.594 ± 0.013	2.585 ± 0.020
PROS1	Vitamin K-dependent protein S	0.831 ± 0.022	0.951 ± 0.007	2.458 ± 0.062	2.449 ± 0.043
APOA1	Apolipoprotein A-I	1.484 ± 0.018	1.511 ± 0.003	2.343 ± 0.016	2.228 ± 0.041
PIGR	Polymeric immunoglobulin receptor	6.355 ± 0.044	8.625 ± 0.078	7.088 ± 0.061	8.362 ± 0.087
PLG	Plasminogen	6.697 ± 0.110	8.641 ± 0.093	7.023 ± 0.227	6.802 ± 0.141
THBS1	Thrombospondin-1	0.354 ± 0.006	0.400 ± 0.004	0.981 ± 0.025	0.966 ± 0.004
SERPINC1	Antithrombin-III	1.704 ± 0.005	1.957 ± 0.020	1.882 ± 0.019	1.561 ± 0.017
C1S	Complement C1s subcomponent	0.377 ± 0.006	0.441 ± 0.007	1.034 ± 0.008	1.060 ± 0.029
AHSG	Alpha-2-HS-glycoprotein	4.642 ± 0.039	5.371 ± 0.089	3.468 ± 0.025	3.677 ± 0.041
C9	Complement component C9	0.520 ± 0.006	0.613 ± 0.010	1.512 ± 0.053	1.633 ± 0.036
SERPINF1	Pigment epithelium-derived factor	0.076 ± 0.005	0.081 ± 0.000	0.349 ± 0.015	0.370 ± 0.015
A1BG	Alpha-1B-glycoprotein	0.633 ± 0.011	0.807 ± 0.002	1.940 ± 0.017	1.946 ± 0.024
ORM1	Alpha-1-acid glycoprotein	1.881 ± 0.096	2.267 ± 0.139	0.501 ± 0.020	0.510 ± 0.031
SERPINA3-2	Serpin A3-2	0.627 ± 0.006	0.626 ± 0.004	0.351 ± 0.003	0.347 ± 0.009
F9	Coagulation factor IX	1.363 ± 0.006	1.496 ± 0.014	2.481 ± 0.016	2.738 ± 0.021
APOA4	Apolipoprotein A-IV	0.893 ± 0.018	0.848 ± 0.006	1.716 ± 0.003	1.709 ± 0.011
GSN	Gelsolin	0.160 ± 0.005	0.171 ± 0.002	0.336 ± 0.019	0.379 ± 0.008
HBB	Hemoglobin subunit beta	0.761 ± 0.004	0.876 ± 0.005	1.664 ± 0.016	1.697 ± 0.019
APOH	Beta-2-glycoprotein 1	2.710 ± 0.016	2.315 ± 0.046	3.032 ± 0.013	2.713 ± 0.026
HPX	Hemopexin	0.578 ± 0.011	0.711 ± 0.010	0.442 ± 0.002	0.473 ± 0.003
APOH	Beta-2-glycoprotein 1	2.710 ± 0.016	2.315 ± 0.046	3.032 ± 0.013	2.713 ± 0.026
HPX	Hemopexin	0.578 ± 0.011	0.711 ± 0.010	0.442 ± 0.002	0.473 ± 0.003

[1]The percentage was calculated based on all identified proteins (57 proteins) by LC-MS
[2]Data were analysed using bovine database.
[3]Values are expressed as mean ± SD (n=3).

Table S4. Quantitative analysis of proteins of FBS-incubated MSC EV samples (continued).

Gene code	Description	Average in %[1,2,3] EV_{3D-1}	EV_{3D-2}	EV_{2D-1}	EV_{2D-2}
	Factor XIIa inhibitor	0.345 ± 0.002	0.393 ± 0.006	0.690 ± 0.013	0.708 ± 0.017
LBP	Lipopolysaccharide-binding protein	0.396 ± 0.003	0.411 ± 0.003	1.336 ± 0.016	1.276 ± 0.026
C1QB	Complement C1q subcomponent subunit B	1.119 ± 0.026	1.245 ± 0.019	1.833 ± 0.045	1.920 ± 0.015
	Hemoglobin fetal subunit beta	1.213 ± 0.005	1.313 ± 0.011	2.956 ± 0.021	2.896 ± 0.037
GC	Vitamin D-binding protein	2.423 ± 0.083	0.892 ± 0.025	0.743 ± 0.021	0.757 ± 0.072
PGLYRP1	Peptidoglycan recognition protein 1	1.280 ± 0.015	1.124 ± 0.016	1.720 ± 0.014	1.203 ± 0.014
MBL	Mannose-binding protein C	0.692 ± 0.002	0.862 ± 0.002	1.302 ± 0.011	1.206 ± 0.019
HBA	Hemoglobin subunit alpha	0.983 ± 0.003	1.023 ± 0.025	1.044 ± 0.010	1.189 ± 0.014
AFP	Alpha-fetoprotein	0.421 ± 0.005	0.253 ± 0.003	0.254 ± 0.001	0.273 ± 0.002
APOE	Apolipoprotein E	0.196 ± 0.005	0.235 ± 0.002	0.427 ± 0.005	0.506 ± 0.016
C4	Complement C4 (Fragments)	0.188 ± 0.001	0.207 ± 0.002	0.595 ± 0.012	0.619 ± 0.008
C1QA	Complement C1q subcomponent subunit A	0.466 ± 0.014	0.416 ± 0.004	1.204 ± 0.017	1.274 ± 0.034
PSMB9	Proteasome subunit beta type-9	0.038 ± 0.002	0.037 ± 0.001	0.075 ± 0.001	0.077 ± 0.002
SERPINA3-5	Serpin A3-5	0.257 ± 0.001	0.306 ± 0.004	0.527 ± 0.016	0.489 ± 0.020
GPX3	Glutathione peroxidase 3	0.044 ± 0.001	0.048 ± 0.002	0.145 ± 0.003	0.161 ± 0.003
NFIL3	Nuclear factor interleukin-3-regulated protein	1.831 ± 0.013	1.552 ± 0.035	1.743 ± 0.024	1.493 ± 0.061
URAD	2-oxo-4-hydroxy-4-carboxy-5-ureidoimidazoline decarboxylase	0.223 ± 0.016	0.114 ± 0.006	1.465 ± 0.026	1.564 ± 0.034
ACTB	Actin_ cytoplasmic 1	0.347 ± 0.004	0.357 ± 0.001	0.935 ± 0.011	1.025 ± 0.018
ARL8B	ADP-ribosylation factor-like protein 8B	0.143 ± 0.002	0.173 ± 0.002	0.430 ± 0.004	0.418 ± 0.007
CABP5	Calcium-binding protein 5	1.400 ± 0.040	0.427 ± 0.022	1.454 ± 0.054	1.514 ± 0.151
ASB8	Ankyrin repeat and SOCS box protein 8	0.107 ± 0.002	0.130 ± 0.004	0.458 ± 0.014	0.444 ± 0.003
SERPINA3-8	Serpin A3-8	0.100 ± 0.003	0.120 ± 0.002	0.385 ± 0.015	0.425 ± 0.013
GUCA1A	Guanylyl cyclase-activating protein 1	5.806 ± 0.070	4.621 ± 0.067	5.381 ± 0.063	5.598 ± 0.023
ATP6V1E1	V-type proton ATPase subunit E 1	0.357 ± 0.007	0.403 ± 0.004	1.685 ± 0.015	1.824 ± 0.035
UBB	Polyubiquitin-B	0.095 ± 0.002	0.112 ± 0.003	0.417 ± 0.014	0.439 ± 0.018
FETUB	Fetuin-B	0.053 ± 0.002	0.063 ± 0.001	0.008 ± 0.002	0.008 ± 0.000
LYSMD1	LysM and putative peptidoglycan-binding domain-containing protein 1	0.742 ± 0.011	0.852 ± 0.013	0.799 ± 0.031	0.669 ± 0.039
RAP2C	Ras-related protein Rap-2c	0.309 ± 0.005	0.354 ± 0.006	0.482 ± 0.009	0.504 ± 0.010
RTCA	RNA 3'-terminal phosphate cyclase	0.206 ± 0.005	0.298 ± 0.003	0.633 ± 0.005	0.777 ± 0.013
PCLAF	PCNA-associated factor	0.071 ± 0.005	0.079 ± 0.003	0.215 ± 0.007	0.217 ± 0.021

[1]The percentage was calculated based on all identified proteins (57 proteins) by LC-MS

[2]Data were analysed using bovine database.

[3]Values are expressed as mean ± SD (n=3).

Table S5. Doses applied for *in vitro* and *in vivo* studies.

Samples	*In vitro* dose		*In vivo* dose			
			Optical imaging		Flow cytometry	
	FI[1]	Particle per well[2]	FI[3]	Particles per mouse	FI[4]	Particles per mouse
EV_{2D}	≈150	$2x10^9$	≈$5.25x10^8$	≈$2x10^{11}$	≈2500	≈$2x10^{11}$
EV_{3D}	≈150	$2x10^9$	≈$5.25x10^8$	≈$2x10^{11}$	≈2500	≈$2x10^{11}$
HC-EV_{2D}	≈150	$2x10^9$				
HC-EV_{3D}	≈150	$2x10^9$				

[1] Fluorescence intensity (FL) obtained for a 100 µl sample measured by FLUOstar® Omega plate reader (excitation at 499 nm, emission at 520 nm) expressed in arbitrary units.
[2]The experiment was performed in 24-well plate
[3]FL obtained for a 100 µl samples measured by IVIS® optical imaging (excitation at 780 nm, emission at 840 nm) expressed in total Radiant Efficiency [p/s] / [µW/cm²]
[4] Fluorescence intensity (FL) obtained for a 100 µl sample measured by FLUOstar® Omega plate reader (excitation at 644 nm, emission at 663 nm) expressed in arbitrary units.

Table S6. Non-incubated MSC EV proteins and their association with cellular components.

Cellular component	Mapped gene names (UniProt)
Extracellular space	P68133; P60709; P02765; P01023; P04083; P07355; O43866; O00299; P10909; P01024; P02751; Q08380; P06396; P00738; P11142; P69905; P01876; P01860; P01871; Q08431; P0CG47; A5A3E0; P00734; P02787; P02768; O00560; P07996; P04004
Cytoplasm	P60709; P04083; P07355; P08758; O43866; O00299; P10909; P06396; P04150; P04406; P17066; P11142; Q14568; Q9NVM9; Q8IU60; P41219; P0CG47; P14618; P02768; O00560
Plasma membrane	P60709; P04083; P07355; O43866; O00299; P01024; P02751; P06396; P04406; P17066; P11142; P01871; P08514; P54105; P0CG47; P00734; P02787; O00560
Nucleus	P60709; P04083; P07355; O00299; P10909; P04150; P04406; P17066; P11142; Q9NVM9; P54105; P0CG47; P14618; P02768; O00560

Table S7. Biological processes hypothesised to contribute to MSC EV clearance based on extracellular space protein analysis identified by LC-MS analysis.

Biological process	EV proteins	
	Mapped gene names (UniProt)	Protein name
Opsonisation	P01024	Complement C3
Positive regulation of opsonization	P01024	Complement C3
Complement activation (classical pathway),	P10909; P01024; P01876; P01860; P01871;	Clusterin, Complement C3, Immunoglobulin heavy constant alpha 1, Immunoglobulin heavy constant gamma 3, Immunoglobulin heavy constant mu
Complement activation (alternative pathway)	P01024	Complement C3
Phagocytosis, engulfment	P06396; P01876; P01860; P01871; Q08431	Gelsolin, Immunoglobulin heavy constant alpha 1, Immunoglobulin heavy constant gamma 3, Immunoglobulin heavy constant mu, Lactadherin
Positive regulation of phagocytosis	P02765; Q08431	Alpha-2-HS-glycoprotein, Lactadherin

Table S8. Biological processes hypothesised to contribute to MSC EVs clearance based on protein corona composition analysis identified by LC-MS analysis.

Biological process	Protein corona compositions	
	Mapped gene names (UniProt)	Protein name
Positive regulation of opsonisation	O02659	Mannose-binding protein C
Complement activation (classical pathway),	Q5E9E3; Q2KIV9; Q0VCX1; Q2UVX4; P01030; Q29RQ1; Q3MHN2; O02659	Complement C1q subcomponent subunit A, Complement C1q subcomponent subunit B, Complement C1s subcomponent, Complement C3, Complement C4, Complement component C7, Complement component C9, Mannose-binding protein C
Complement activation (alternative pathway)	Q2UVX4; Q29RQ1; Q3MHN2; P81187	Complement C3, Complement component C7, Complement component C9, Complement factor B
Complement activation (lectin pathway)	O02659	Mannose-binding protein C
Positive regulation of phagocytosis	P12763; P15497; O02659	Alpha-2-HS-glycoprotein, Apolipoprotein A-IV, Mannose-binding protein C

Table S9. Distribution of typical plasma protein receptors in liver sub-populations.

Proteins (Gene code)	Receptors	Cell expression	References
ALB	gp18, gp30, gp60	Hepatocytes	[3], [4], [5]
		Endothelial cells	[3], [4]
	Fc Rn	Hepatocytes	[4], [6], [7]
		Endothelial cells	[4], [6]
		Kupffer cells	[4], [7]
		Stellate cells	[4], [8]
	SPARC	Endothelial cells	[9]
		Stellate cells	[8]
	TGF-β	Hepatocytes	[10]
		Stellate cells	[8], [10]
ORM1, AHSG	ASGP-R	Hepatocytes	[11], [12], [13], [14]
SERPINA1<br>A2M, A2M<br>SERPINA3-2<br>SERPINA3-7<br>SERPINC1	Serpin	Hepatocytes	[15], [16]
A1BG	Lectin	Hepatocytes	[17]
APOA1<br>APOA4	Apolipoprotein	Hepatocytes	[7], [18], [19]
		Kupffer cells	[7]
APOE	Apolipoprotein E	Hepatocytes	[7], [20]
		Kupffer cells	[7], [21]
		Endothelial cells	[21]
HSPA6<br>HSP90AA2P	CD91	Hepatocytes	[22], [23], [24]
		Kupffer cells	
HBG2, HBA, HBB, HPX	Hb/Hp	Hepatocytes	[25], [26]
PLG	tPA	Hepatocytes	[27], [28], [29]
		Kupffer cells	[27], [28]
		Endothelial cells	[27], [28]
TF	TfR2	Hepatocytes	[7]
AHSG<br>MBL	Scavenger (SR-A)	Kupffer cells	[13], [30]
		Endothelial cells	[13]
LTF	Mannose	Kupffer cells	[30], [8], [31]
		Endothelial cells	[8], [31]
C1QA, C1QB, C1S, C3, C4, C7, C9, CFB	Complement	Kupffer cells	[8], [32]
VTN	αVβ3	Kupffer cells	[33], [34]
		Endothelial cells	[33], [34]
		Stellate cells	[8], [33], [34]

Supplementary References

1. Saleh, Amer F et al., Extracellular vesicles induce minimal hepatotoxicity and immunogenicity. Nanoscale vol. 11,14 (2019): p. 6990-7001.
2. Smyth, Tyson et al., Surface functionalization of exosomes using click chemistry. Bioconjugate chemistry vol. 25,10 (2014): p. 1777-84.
3. Schnitzer, J.E., et al., Preferential interaction of albumin-binding proteins, gp30 and gp18, with conformationally modified albumins. Presence in many cells and tissues with a possible role in catabolism. J Biol Chem, 1992. 267(34): p. 24544-53.
4. Bernardi, M., et al., Albumin in decompensated cirrhosis: new concepts and perspectives. Gut, 2020. 69(6): p. 1127-1138.
5. Mocan, L., et al., Photothermal treatment of liver cancer with albumin-conjugated gold nanoparticles initiates Golgi Apparatus-ER dysfunction and caspase-3 apoptotic pathway activation by selective targeting of Gp60 receptor. Int J Nanomedicine, 2015. 10: p. 5435-45.
6. Latvala, S., et al., Distribution of FcRn Across Species and Tissues. J Histochem Cytochem, 2017. 65(6): p. 321-333.
7. Li, H., et al., The protein corona and its effects on nanoparticle-based drug delivery systems. Acta Biomater, 2021. 129: p. 57-72.
8. Poelstra, K., Innovative Nanotechnological Formulations to Reach the Hepatic Stellate Cell. Current Tissue Microenvironment Reports, 2020. 1(2): p. 13-22.
9. Larsen, M.T., et al., Albumin-based drug delivery: harnessing nature to cure disease. Molecular and Cellular Therapies, 2016. 4(1): p. 3.
10. Ozaki, I., et al., Regulation of TGF-?1-Induced Pro-Apoptotic Signaling by Growth Factor Receptors and Extracellular Matrix Receptor Integrins in the Liver. Frontiers in physiology, 2011. 2: p. 78.
11.Yang, S., et al., Drug delivery strategy in hepatocellular carcinoma therapy. Cell Commun Signal, 2022. 20(1): p. 26.
12. Fan, N., et al., Celastrol-loaded lactosylated albumin nanoparticles attenuate hepatic steatosis in non-alcoholic fatty liver disease. Journal of Controlled Release, 2022. 347: p. 44-54.
13. Herrmann, M., et al., Clearance of Fetuin-A–Containing Calciprotein Particles Is Mediated by Scavenger Receptor-A. Circulation Research, 2012. 111(5): p. 575-584.
14. Matsumoto, K., et al., Receptor-mediated uptake of human alpha1-acid glycoprotein into liver parenchymal cells in mice. Drug Metab Pharmacokinet, 2010. 25(1): p. 101-7.
15. Pizzo, S.V., Serpin receptor 1: A hepatic receptor that mediates the clearance of antithrombin III-proteinase complexes. The American Journal of Medicine, 1989. 87(3, Supplement 2): p. S10-S14.
16. Maekawa, H. and D.M. Tollefsen, Role of the Proposed Serpin-Enzyme Complex Receptor Recognition Site in Binding and Internalization of Thrombin-Heparin Cofactor II Complexes by Hepatocytes*. Journal of Biological Chemistry, 1996. 271(31): p. 18604-18609.
17. McFarlane, I.G., et al., Identification of the hepatic asialo-glycoprotein receptor (hepatic lectin) as a component of liver specific membrane lipoprotein (LSP). Clinical and experimental immunology, 1984. 55(2): p. 347-354.
18. Felmlee, D., et al., Hepatitis C Virus, Cholesterol and Lipoproteins — Impact for the Viral Life Cycle and Pathogenesis of Liver Disease. Viruses, 2013. 5: p. 1292-1324.
19. Dvorin, E., et al., Apolipoprotein A-IV. A determinant for binding and uptake of high density lipoproteins by rat hepatocytes. J Biol Chem, 1986. 261(33): p. 15714-8.
20. Sato, Y., et al., Different kinetics for the hepatic uptake of lipid nanoparticles between the apolipoprotein E/low density lipoprotein receptor and the N-acetyl-d-galactosamine/asialoglycoprotein receptor pathway. Journal of Controlled Release, 2020. 322: p. 217-226.
21. Schouten, D., et al., Interaction in vivo and in vitro of apolipoprotein E-free high-density lipoprotein with parenchymal, endothelial and Kupffer cells from rat liver. Biochemical Journal, 1988. 256(2): p. 615-621.
22. Basu, S., et al., CD91 Is a Common Receptor for Heat Shock Proteins gp96, hsp90, hsp70, and Calreticulin. Immunity, 2001. 14(3): p. 303-313.

23. Zhao, D., et al., ALK1 signaling is required for the homeostasis of Kupffer cells and prevention of bacterial infection. J Clin Invest, 2022. 132(3).
24. Takemoto, S., M. Nishikawa, and Y. Takakura, Pharmacokinetic and tissue distribution mechanism of mouse recombinant heat shock protein 70 in mice. Pharm Res, 2005. 22(3): p. 419-26.
25. Higa, Y., et al., Catabolism of globin-haptoglobin in liver cells after intravenous administration of hemoglobin-haptoglobin to rats. Journal of Biological Chemistry, 1981. 256(23): p. 12322-12328.
26. Gburek, J., et al., Hemoglobin - a novel ligand of hepatocyte ectopic F1-ATPase. J Physiol Pharmacol, 2015. 66(6): p. 823-30.
27. Redlitz, A. and E.F. Plow, Receptors for plasminogen and t-PA: an update. Baillieres Clin Haematol, 1995. 8(2): p. 313-27.
28. Okada, K., et al., Binding of plasminogen to hepatocytes isolated from injured mice liver and nonparenchymal cell-dependent proliferation of hepatocytes. Blood Coagulation & Fibrinolysis, 2008. 19(6).
29. Okumura, N., et al., A Novel Function of Thrombin-activatable Fibrinolysis Inhibitor during Rat Liver Regeneration and in Growth-promoted Hepatocytes in Primary Culture*. Journal of Biological Chemistry, 2009. 284(24): p. 16553-16561.
30. Ono, K., et al., Mannose-Binding Lectin Augments the Uptake of Lipid A, Staphylococcus aureus, and Escherichia coli by Kupffer Cells through Increased Cell Surface Expression of Scavenger Receptor A1. The Journal of Immunology, 2006. 177.
31. Zlatina, K. and S.P. Galuska, The N-glycans of lactoferrin: more than just a sweet decoration. Biochemistry and Cell Biology, 2020. 99(1): p. 117-127.
32. Helmy, K.Y., et al., CRIg: A Macrophage Complement Receptor Required for Phagocytosis of Circulating Pathogens. Cell, 2006. 124(5): p. 915-927.
33. Fadok, V.A., et al., Different populations of macrophages use either the vitronectin receptor or the phosphatidylserine receptor to recognize and remove apoptotic cells. J Immunol, 1992. 149(12): p. 4029-35.
34. Patsenker, E., et al., Pharmacological inhibition of integrin alphavbeta3 aggravates experimental liver fibrosis and suppresses hepatic angiogenesis. Hepatology (Baltimore, Md.), 2009. 50(5): p. 1501-1511.
