# Supplementary material for: Cellular uptake and in vivo distribution of mesenchymal-stem-cell-derived extracellular vesicles are protein corona dependent
Source: Nat Nanotechnol. 2024 Feb 16;19(6):846–55. doi: 10.1038/s41565-023-01585-y (PMC11186763; doi:10.1038/s41565-023-01585-y)
Supplement: Supplementary file 2 — Reporting Summary [file 41565_2023_1585_MOESM2_ESM.pdf]

nature portfolio

Corresponding author(s): Khuloud Al-Jamal

Last updated by author(s): Oct 30, 2023

Reporting Summary

Nature Portfolio wishes to improve the reproducibility of the work that we publish. This form provides structure for consistency and transparency in reporting. For further information on Nature Portfolio policies, see our Editorial Policies and the Editorial Policy Checklist.

Statistics

For all statistical analyses, confirm that the following items are present in the figure legend, table legend, main text, or Methods section.

n/a	Confirmed	
☐	☒	The exact sample size (*n*) for each experimental group/condition, given as a discrete number and unit of measurement
☐	☒	A statement on whether measurements were taken from distinct samples or whether the same sample was measured repeatedly
☐	☒	The statistical test(s) used AND whether they are one- or two-sided<br>*Only common tests should be described solely by name; describe more complex techniques in the Methods section.*
☒	☐	A description of all covariates tested
☐	☒	A description of any assumptions or corrections, such as tests of normality and adjustment for multiple comparisons
☐	☒	A full description of the statistical parameters including central tendency (e.g. means) or other basic estimates (e.g. regression coefficient) AND variation (e.g. standard deviation) or associated estimates of uncertainty (e.g. confidence intervals)
☐	☒	For null hypothesis testing, the test statistic (e.g. *F*, *t*, *r*) with confidence intervals, effect sizes, degrees of freedom and *P* value noted<br>*Give P values as exact values whenever suitable.*
☒	☐	For Bayesian analysis, information on the choice of priors and Markov chain Monte Carlo settings
☒	☐	For hierarchical and complex designs, identification of the appropriate level for tests and full reporting of outcomes
☒	☐	Estimates of effect sizes (e.g. Cohen's *d*, Pearson's *r*), indicating how they were calculated

Our web collection on statistics for biologists contains articles on many of the points above.

Software and code

Policy information about availability of computer code

Data collection	Dot blot data were collected by Image Lab software V4.1 (Bio-Rad, UK). Size and concentration of EV data were collected by Nanosight NTA 3.2 software (Malvern Instruments, UK). Zeta potential of EV data were collected by Zetasizer version 7.12 (Malvern Instruments, UK). Data for microBCA were collected by Omega (BMG LABTECH). Flow cytometry data were collected by BD FACStation™ software V6.0 and BD FACSDiva Software V9.2 (BD Biosciences, USA). In vivo and ex vivo imaging data were collected by Living Image® V4.7.3 Software (PerkinElmer, USA).
Data analysis	Microplate reader data analysis was performed through MARS software V2.40. Flow cytometry data analysis was performed by FlowJo v.10.7.2 (TreeStar/BD Bioscience). Proteomic analysis was performed by MassLynx 4.1. Progenesis® QI for Proteomics Software Version 2.0 (using human and bovine UniProt database). Multivariate analysis was performed by Minitab V20 and The Unscrambler X. Bioinformatic analysis was performed by FunRich 3.1.3. Statistical analyses of the data were performed using Prism 9.4.1. MATLAB 9.11 was used to generate heatmaps of data.

For manuscripts utilizing custom algorithms or software that are central to the research but not yet described in published literature, software must be made available to editors and reviewers. We strongly encourage code deposition in a community repository (e.g. GitHub). See the Nature Portfolio guidelines for submitting code & software for further information.

Data

Policy information about availability of data

All manuscripts must include a data availability statement. This statement should provide the following information, where applicable:

- Accession codes, unique identifiers, or web links for publicly available datasets
- A description of any restrictions on data availability
- For clinical datasets or third party data, please ensure that the statement adheres to our policy

UniProtKB/SwissProt Homo sapiens and Bos taurus fasta database (Proteome ID: UP000005640 and UP000009136, respectively) were used in this study. The authors declare that all data supporting the findings of this study are available in provided source data and supplementary information. Additional data are also available from the corresponding author upon reasonable request.

Research involving human participants, their data, or biological material

Policy information about studies with human participants or human data. See also policy information about sex, gender (identity/presentation), and sexual orientation and race, ethnicity and racism.

Reporting on sex and gender	N/A
Reporting on race, ethnicity, or other socially relevant groupings	N/A
Population characteristics	N/A
Recruitment	N/A
Ethics oversight	N/A

Note that full information on the approval of the study protocol must also be provided in the manuscript.

Field-specific reporting

Please select the one below that is the best fit for your research. If you are not sure, read the appropriate sections before making your selection.

☒ Life sciences ☐ Behavioural & social sciences ☐ Ecological, evolutionary & environmental sciences

For a reference copy of the document with all sections, see nature.com/documents/nr-reporting-summary-flat.pdf

Life sciences study design

All studies must disclose on these points even when the disclosure is negative.

Sample size	For in vitro experiments, three biological replicates for treatment were performed. For in vivo experiments, a group size of 3 (mice) is the minimum requirement for statistical analysis for biodistribution. No statistical methods were used to pre-determine sample sizes, but the sample sizes were chosen based on the prior experiments similarly performed by our group with proven statistically significant effects (PMID 30144166 and 33470092)
Data exclusions	There are no data exclusions.
Replication	All assays were performed with three biological replicates and/or at least two technical replicates. All attempts at replication were successful.
Randomization	For animal experiments, healthy mice were randomly assigned into groups.
Blinding	All experiments often involve quantifiable endpoints, such as fluorescence intensity and other quantitative methods. These measurements are less susceptible to bias because they are typically analyzed using instruments or software, reducing the impact of human subjectivity.

Reporting for specific materials, systems and methods

We require information from authors about some types of materials, experimental systems and methods used in many studies. Here, indicate whether each material, system or method listed is relevant to your study. If you are not sure if a list item applies to your research, read the appropriate section before selecting a response.

Materials & experimental systems

n/a	Involved in the study
☐	☒ Antibodies
☐	☒ Eukaryotic cell lines
☒	☐ Palaeontology and archaeology
☐	☒ Animals and other organisms
☒	☐ Clinical data
☒	☐ Dual use research of concern
☒	☐ Plants

Methods

n/a	Involved in the study
☒	☐ ChIP-seq
☐	☒ Flow cytometry
☒	☐ MRI-based neuroimaging

Antibodies

Antibodies used

Mouse anti-human CD81 antibody (clone 5A6, BioLegend, cat#349502), Mouse anti-human CD9 antibody (clone HI9a, BioLegend, cat#312102), Rabbit anti- human CD63 antibody (clone EPR5702
, Abcam, cat#ab134045), TSG101 polyclonal antibody (Proteintech, cat#14497-1-AP), HRP-goat anti-rabbit antibody (Cell Signalling Technologies, cat#7074S), HRP-goat anti-mouse antibody (Cell Signalling Technologies, cat#7076S), Anti- mouse CD45 antibody (PerCP) (clone 30-F11, BioLegend, cat#103129), Anti-mouse CD45 antibody (PE) (clone 30-F11, BioLegend, cat#103105), Anti-mouse F4/80 antibody (FITC) (clone BM8, BioLegend, cat#123107), Anti-mouse CD11b antibody (PerCP) (clone M1/70, BioLegend, cat#101229), Anti-mouse CD31 antibody (PE) (clone 390, BioLegend, cat#102507), Anti-ASGPR1 antibody - Alexa Fluor® 647 (clone 8D7, Santa Cruz Biotechnology, cat#sc-52623), Anti-human/ mouse/rat GFAP, REAfinityTM (Miltenyi Biotec, cat#130-124-040), Anti-mouse CD146-FITC antibody (clone ME9F1, Miltenyi Biotec, cat#130-102-230), Mouse SPARC Alexa Fluor 647-conjugated Antibody (clone 124413, R&D systems, cat# IC942R)

Validation

Mouse anti-human CD81 antibody has been validated by the manufacturer (BioLegend) to be specific to human species and was quality control tested by immunofluorescent staining with flow cytometric analysis. Application in WB and IP are reported in the literature (PMID: 14966136)

Mouse anti-human CD9 antibody has been validated by the manufacturer (BioLegend) to be specific to human species and was quality control tested by immunofluorescent staining with flow cytometric analysis. Application in dot blot is reported in the literature (PMID: 33368666)

Rabbit anti- human CD63 antibody has been validated by the manufacturer (Abcam) to be specific to human species and was verified for application in WB and IHC-P.

TSG101 polyclonal antibody has been validated by the manufacturer (Proteintech) to be specific to human, mouse, and rat species and was verified for application in WB, RIP, IP, IHC, IF, FC, and ELISA.

HRP-goat anti-rabbit antibody and HRP-goat anti-mouse antibody were thoroughly validated by the manufacturer (Cell Signalling Technologies) with CST primary antibodies and will work optimally with the CST western immunoblotting protocol, ensuring accurate and reproducible results.

Anti- mouse CD45 antibody (PerCP), Anti-mouse CD45 antibody (PE), Anti-mouse F4/80 antibody (FITC), Anti-mouse CD11b antibody, and Anti-mouse CD31 antibody (PE) has been validated by the manufacturer (BioLegend) to be specific to mouse species and were quality control tested by immunofluorescent staining with flow cytometric analysis.

ASGPR1 antibody has been validated by the manufacturer (Santa Cruz Biotechnology) to be used for detecting ASGPR1 of mouse, rat and human origin by WB, IP, IF and FCM (cited in 12 publications)

GFAP antibody has been validated by the manufacturer (Miltenyi Biote) to be used for detecting GFAP of human/ mouse/rat by ICFC, MICS, 3D-IF, IF, and IHC.

CD146-FITC antibody has been validated by the manufacturer (Miltenyi Biote) to be used for detecting CD146 of mouse by FC, MICS, IF, and IHC.

SPARC Alexa Fluor® 647-conjugated Antibody has been validated by the manufacturer (R&D systems) to be used for detecting SPARC/ Osteonectin of mouse by intracellular staining by flow cytometry.

Eukaryotic cell lines

Policy information about cell lines and Sex and Gender in Research

Cell line source(s)	HepG2 (human hepatocellular carcinoma, ATCC® HB-8065) and J774A.1 (BALB/c mouse macrophage, ATCC®TIB-67™) were purchased from American Type Culture Collection (ATCC).
Authentication	The cell lines used in the study were not authenticated.
Mycoplasma contamination	All the cell lines were tested negative for mycoplasma contamination.

Commonly misidentified lines (See ICLAC register)	No commonly misidentified lines were used.

Animals and other research organisms

Policy information about studies involving animals; ARRIVE guidelines recommended for reporting animal research, and Sex and Gender in Research

Laboratory animals	5-week-old CD-1 mice (Charles River, UK)<br>6-8- week-old C57BL/6 mice (Charles River, UK)<br>Mice were housed in 12h light/12h dark cycle with the temperature maintained between 65-75°F (~18-23°C), ~50% humidity.
Wild animals	No wild animals were used.
Reporting on sex	Sex of animals were not considered in the design of this study.
Field-collected samples	No field-collected samples were used.
Ethics oversight	All animal experiments were performed in compliance with the UK Animals (Scientific Procedures) Act 1986 and UK Home Office Code of Practice for the Housing and Care of Animals Used in Scientific Procedures (Home Office 1989). In vivo experimentation was adhered to the project license approved by the King's College London animal welfare and ethical review body (AWERB) and UK Home Office (PBE6EB195 and PP8950634). Animal research and veterinary care was performed at Franklin-Wilkins Building, King's College London under the protocol approved for this study by Named Training and Competency Officer (NTCO, Julie Keeble) and Named Animal Care and Welfare Officer (NACWO, Jayne Morgan).

Note that full information on the approval of the study protocol must also be provided in the manuscript.

Flow Cytometry

Plots

Confirm that:

☒ The axis labels state the marker and fluorochrome used (e.g. CD4-FITC).

☒ The axis scales are clearly visible. Include numbers along axes only for bottom left plot of group (a 'group' is an analysis of identical markers).

☒ All plots are contour plots with outliers or pseudocolor plots.

☒ A numerical value for number of cells or percentage (with statistics) is provided.

Methodology

Sample preparation	In the flow cytometric analysis, Alexa-488 labelled EVs were used to track the cellular uptake in vitro. After treating different cell types with fluorescently labelled EVs for 1h, 4h, and 24h, the cells were washed, trypsinised, and collected for centrifugation at 600 g for 5 min, followed by washing with PBS twice. The suspended cells were subjected to flow cytometry analysis. For in vivo uptake investigation, DiD labelled EVs were used. After 24-h IV administration, the liver was perfused, digested, and strained using 70 µM cell strainer. Liver subpopulations were then separated using differential centrifugation. The cells were then stained with specific markers for characterisation and to identify the colocalised signals of each cell type and labelled EVs to determine the cellular uptake.
Instrument	BD FACSCalibur™ and BD FACSCelesta™ (BD Biosciences, USA)
Software	Data were acquired using BD FACStation™ software v6.0 and BD FACSDiva Software v9.2.
Cell population abundance	All flow data were acquired from at least 10000 gated for live populations.
Gating strategy	Cells were first gated using FSC-A and SSC-A. The singlet population was acquired using FSC-H and FSC-A. Aqua (viability dye) negative population was gated, followed by gating based on cell marker expression used to characterise each liver subpopulation.

☒ Tick this box to confirm that a figure exemplifying the gating strategy is provided in the Supplementary Information.
