# Supplementary material for: Cellular uptake and in vivo distribution of mesenchymal-stem-cell-derived extracellular vesicles are protein corona dependent
Source: Nat Nanotechnol. 2024 Feb 16;19(6):846–55. doi: 10.1038/s41565-023-01585-y (PMC11186763; doi:10.1038/s41565-023-01585-y)
Supplement: Supplementary file 4 — Numerical source data and uncropped dot-blots, respectively. [file 41565_2023_1585_MOESM4_ESM.zip › Uncropped_DotBlots_Fig1A.pdf]

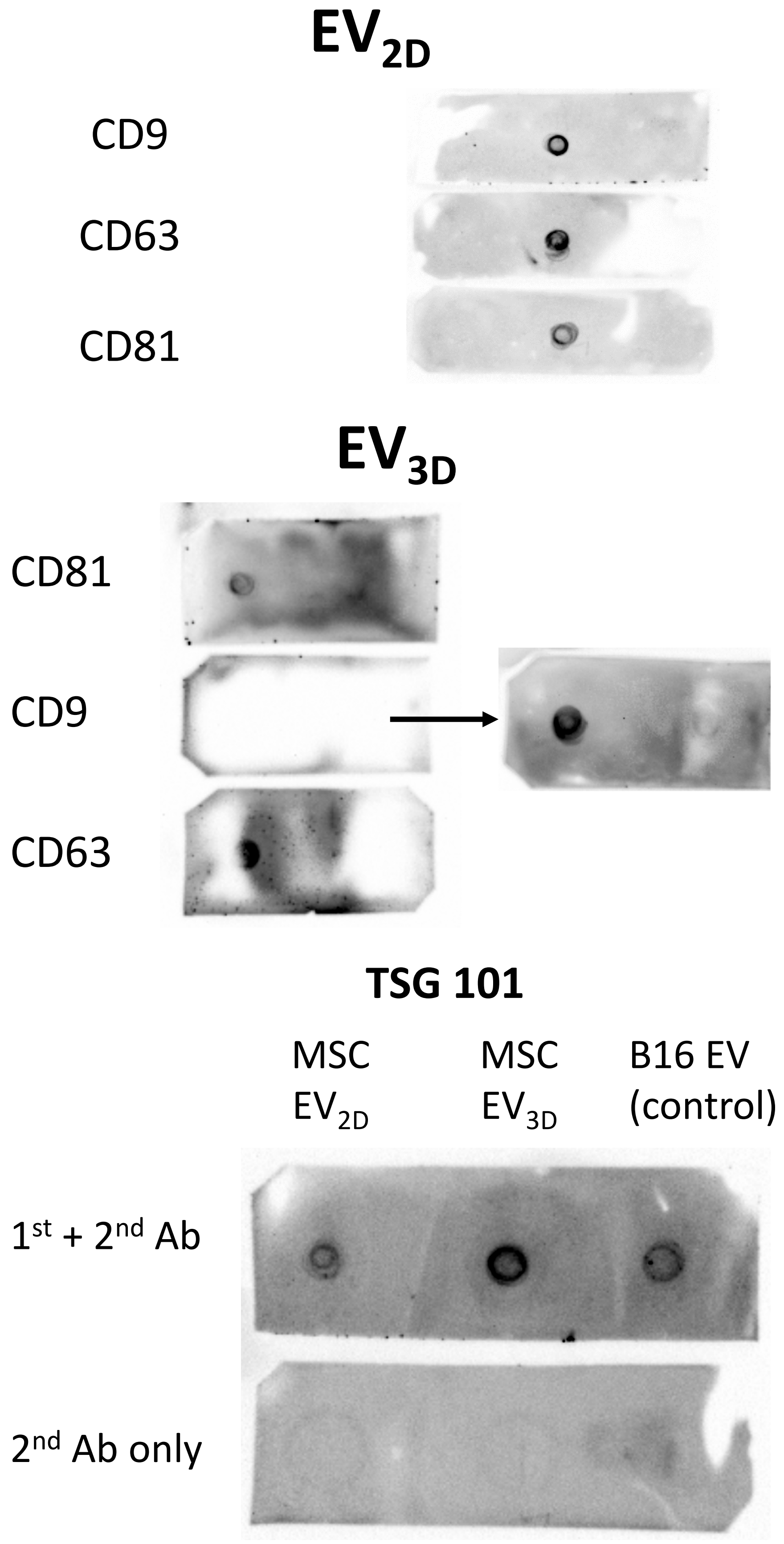

*According to our protocol, EVs were spotted on 1 membrane (per marker) to facilitate the antibody incubation step. The samples derive from parallel experiments and were processed in parallel.
