# Supplementary figures and images for: Cellular uptake and in vivo distribution of mesenchymal-stem-cell-derived extracellular vesicles are protein corona dependent
Source: Nat Nanotechnol. 2024 Feb 16;19(6):846–55. doi: 10.1038/s41565-023-01585-y (PMC11186763; doi:10.1038/s41565-023-01585-y)

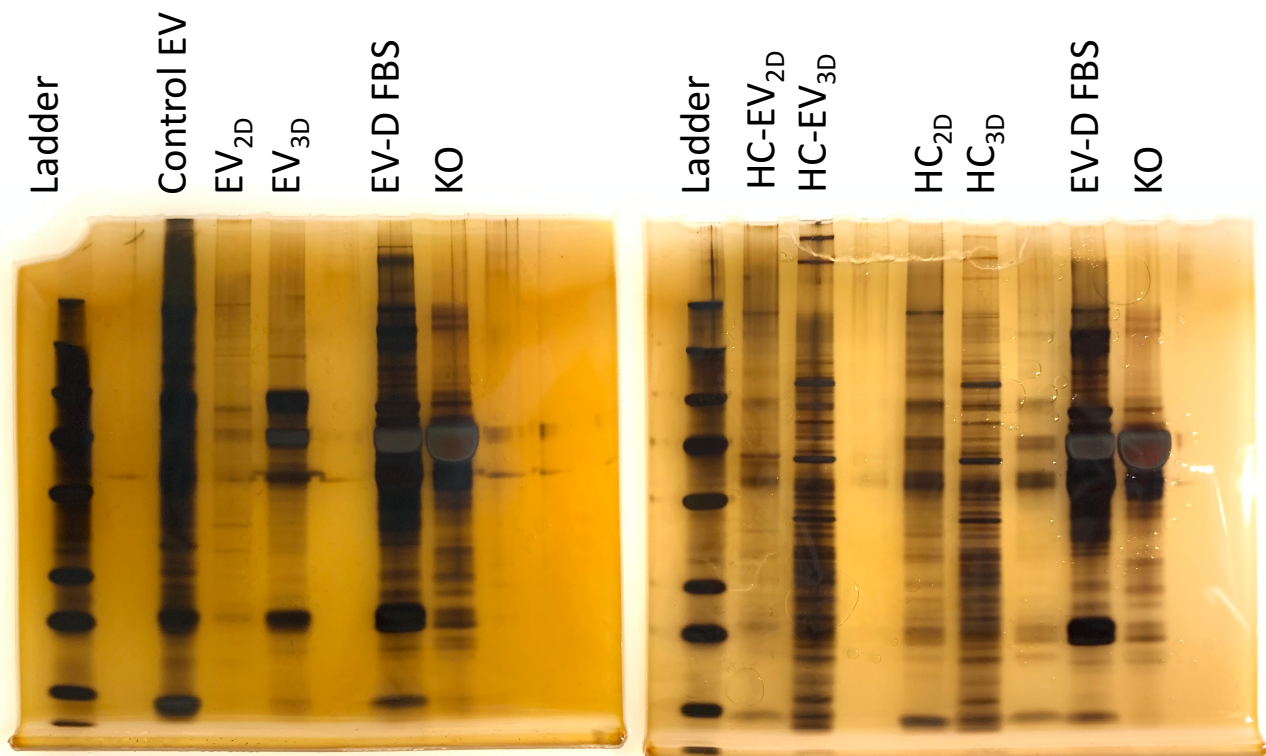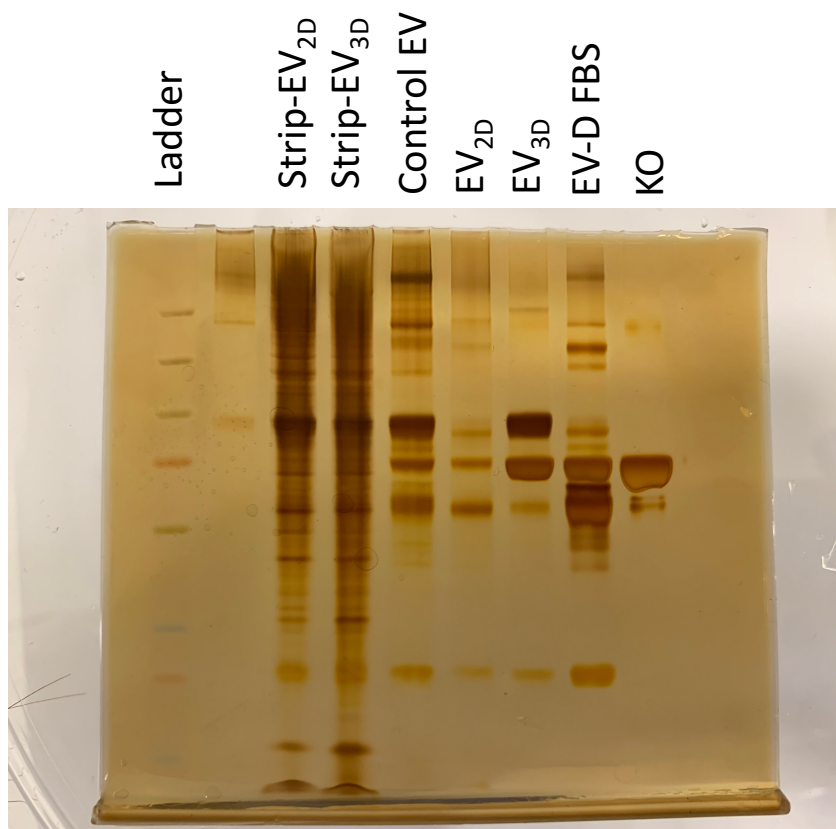

\*The samples derive from parallel experiments and were processed in parallel.

Supplement: Supplementary file 5 — Numerical source data and uncropped gels, respectively. [file 41565_2023_1585_MOESM5_ESM.zip › Uncropped_Gels_Fig2C.pdf]
